# Heavy Metal Stress-Associated Proteins in Rice and *Arabidopsis*: Genome-Wide Identification, Phylogenetics, Duplication, and Expression Profiles Analysis

**DOI:** 10.3389/fgene.2020.00477

**Published:** 2020-05-08

**Authors:** Jiaming Li, Minghui Zhang, Jian Sun, Xinrui Mao, Jingguo Wang, Hualong Liu, Hongliang Zheng, Xianwei Li, Hongwei Zhao, Detang Zou

**Affiliations:** ^1^Key Laboratory of Germplasm Enhancement, Physiology and Ecology of Food Crops in Cold Region, Ministry of Education, Northeast Agricultural University, Harbin, China; ^2^College of Life Science, Northeast Agricultural University, Harbin, China

**Keywords:** *Arabidopsis*, gene duplication, heavy-metal stress, phylogenetic analysis, rice

## Abstract

Heavy metal exposure is a serious environmental stress in plants. However, plants have evolved several strategies to improve their heavy metal tolerance. Heavy metal-associated proteins (HMPs) participate in heavy metal detoxification. Here, we identified 46 and 55 HMPs in rice and *Arabidopsis*, respectively, and named them OsHMP 1–46 and AtHMP 1–55 according to their chromosomal locations. The HMPs from both plants were divided into six clades based on the characteristics of their heavy metal-associated domains (HMA). The HMP gene structures and motifs varied greatly among the different classifications. The HMPs had high collinearity and were segmentally duplicated. A *cis*-element analysis revealed that the HMPs may be regulated by different transcription factors. An expression profile analysis disclosed that only eight OsHMPs were constitutive in rice tissues. Of these, the expression of *OsHMP37* was far higher than that of the other seven genes while *OsHMP28* was expressed exclusively in the roots. For *Arabidopsis*, nine *AtHMP*s presented with very high transcript levels in all organs. Most of the selected *OsHMP*s were differentially expressed in various tissues under different heavy metal stresses. Only *OsHMP09, OsHMP18*, and *OsHMP22* showed higher expression levels in all tissues under different heavy metal stresses. In contrast, most of the selected *AtHMP*s had nearly constant expression levels in different tissues under various heavy metal stresses. The *AtHMP20, AtHMP23, AtHMP25, AtHMP31, AtHMP35, AtHMP46* expression levels under different heavy metal stresses were higher in the leaves and roots. The foregoing discoveries elucidated HMP evolution in monocotyledonous and dicotyledonous plants and may helpful functionally characterize HMPs in the future.

## Introduction

Heavy metal pollution is a serious environmental problem associated with agricultural development and industrialization. It exerts negative effects on plants and threatens human health by causing heavy metal accumulating in food crops (Boyd, 2010). Certain heavy metals such as zinc (Zn) and copper (Cu) are microelements essential for plant growth and metabolism (Yuan et al., 2012). At micro-level concentrations, these heavy metals function as cofactors for enzymes in photosynthesis, biomacromolecule synthesis, electron transport, and other metabolic processes (Ricachenevsky et al., 2013). However, essential heavy metal overaccumulation is toxic to plant cells and inhibits their growth (Thomine and Vert, 2013; Cambrollé et al., 2015). Certain non-essential heavy metals such as cadmium (Cd) and lead (Pb) are phytotoxic at very low concentrations and adversely affect plant growth and development (Hayat et al., 2012; Gill et al., 2013).

Plants have evolved homeostatic mechanisms such as preventing root metal ion uptake and reducing long-distance metal ion transport to increase their tolerance for these substances (Clemens et al., 2002). In plant cells, metal ions can be rendered less harmful by transport, chelation, traffic, and vacuolar sequestration (Hall, 2002). Plants activate various signaling pathways and defense mechanisms that synthesize stress-related proteins in response to heavy metal exposure (Mourato et al., 2015). Heavy metal-associated proteins (HMPs) play key roles in heavy metal transport and detoxification in plant cells. HMPs are metalloproteins or metallochaperone-like proteins containing heavy metal-associated (HMA) domains (Tehseen et al., 2010; Zhang X. D. et al., 2018). The HMA domain is conserved and comprises ~30 amino acid residues. It occurs in several proteins that transport or detoxify heavy metals (Bull and Cox, 1994) and contains two cysteine residues that bind and transfer copper, cadmium, cobalt, zinc, and other heavy metal ions (Gitschier et al., 1998).

As a rule, plant proteins containing HMA domains fall into one of the following groups: HPPs (heavy metal-associated plant proteins), HIPPs (heavy metal-associated isoprenylated plant proteins) (de Abreu-Neto et al., 2013), ATX1-like (Puig et al., 2007), and P1B-ATPase (Pedersen et al., 2012). Previous studies focused mainly on the functions of P1B-type ATPase HMPs. Nine and eight P1B-type ATPase HMPs were identified in rice and *Arabidopsis*, respectively (Pedersen et al., 2012; Zhiguo et al., 2018). Of these, *OsHMA3* was found to control the root-to-shoot Cd translocation rates (Miyadate et al., 2011). *OsHMA4* sequesters Cu in root cell vacuoles and limits Cu transport to the grain and its accumulation there (Huang et al., 2016). In *Arabidopsis*, the Cu-translocating ATPase *AtHMA5* is induced by high Cu levels and causes the efflux of excess Cu from the cytosol to the plasma membrane (Andrés-Colás et al., 2006; Kobayashi et al., 2008). Several ATX1-like metallochaperones have been functionally identified in *Arabidopsis* and rice (Zhang X. D. et al., 2018). A previous study reported that the ATX1-like Cu chaperones ATX1 and CCH in rice, *Arabidopsis*, and soybean transferred Cu to yeast Ccc2 P1B-type ATPase which, in turn, enhanced its antioxidant mechanism (Puig et al., 2007). The HPP and HIPP clades contain the largest number of HMPs but only a few genes in them have been functionally investigated. *AtHIPP3* (*AtHMP52* in the present study) was identified as an upstream controller of stress- and development-related regulatory networks. It is also involved in the salicylate-dependent pathogen response pathway and in flower and seed development (Zschiesche et al., 2015).

Rice (*Oryza sativa* L.) and *Arabidopsis thaliana* L. are research models for monocotyledonous and dicotyledonous plants, respectively. In earlier research, different heavy metal gene families in various species were studied or classified separately (Li et al., 2015; Fang et al., 2016; Zhiguo et al., 2018; Khan et al., 2019). Here, we identified all heavy metal-associated proteins in rice and *Arabidopsis*, including HPP, HIPP, ATX, CCH, CCS, and P1B-ATPase HMPs, by repeated HMM searches *in silico*. We analyzed HMP chromosomal distributions, gene synteny, phylogeny, gene structures, motif compositions, *cis*-elements, expression patterns, and heavy metal stress responses in different tissues of rice and *Arabidopsis*. The aims of this study were to clarify the evolutionary and taxonomic relationships among the heavy metal-associated proteins and identify their expression patterns in different tissues and under various types of metal ion stress. This information may serve as a theoretical basis for the elucidation of the mechanisms of heavy metal tolerance and plant-metal interactions.

## Materials and Methods

### Identification and Sequence Analysis of HMP Family Genes in Rice and *Arabidopsis*

The HMPs of various plant species were identified according to a previously described method (Li et al., 2019). The Hidden Markov Model of the HMA domain (PF00403) was downloaded from the Pfam database (http://pfam.xfam.org/) (El-Gebali et al., 2019). The amino acid, genome, and CDS sequence assemblies were downloaded from the EnsemblPlants database (http://plants.ensembl.org/index.html) (Kersey et al., 2018). Candidate proteins were sought with the HMMSEARCH program (https://www.ebi.ac.uk/Tools/hmmer/search/hmmsearch) based on the Bio-Linux system (Dr. Tracey Timms-Wilson, Centre for Ecology & Hydrology (CEH), Oxfordshire, UK). Only proteins with E-value < 0.01 were selected and they were verified against the Pfam and InterPro databases (http://www.ebi.ac.uk/interpro/) (Mitchell et al., 2019).

The MEME program (http://meme-suite.org/) identified conserved HMP family protein motifs. The HMP family gene structures were displayed using Gene Structure Display Server tools (http://gsds.cbi.pku.edu.cn/) (Hu et al., 2015). The chromosomal locations of the HMP family genes were mapped according to their TIGR numbers onto a rice or *Arabidopsis* linkage map using online tools (Kurata and Yamazaki, 2006; Lamesch et al., 2012). The isoelectric points and molecular weights of the HMP family proteins were estimated with ExPASy (http://expasy.org/) (Artimo et al., 2012). Subcellular localizations of the HMP family proteins were predicted by Cell-PLoc v. 2.0 (http://www.csbio.sjtu.edu.cn/bioinf/Cell-PLoc-2/) (Chou and Shen, 2010).

### Phylogenetic Analysis

A multiple alignment was performed on the HMA domain sequences of various plant species using MEGA v. 7.0 (https://www.megasoftware.net/) (Kumar et al., 2016). Unrooted trees were constructed by the maximum likelihood (ML) method with the following parameters: Poisson correction; pairwise deletion; 1,000 bootstrap replicates.

### Gene Duplication Analysis

Synteny blocks of various plant genomes were downloaded from the Plant Genome Duplication Database (PGDD, http://chibba.agtec.uga.edu/duplication/) (Lee et al., 2013). Duplicated HMP gene pairs were connected by solid lines.

### *Cis*-Element Analysis of HMP Family

HMP family gene promoters were downloaded from the Phytozome database (https://phytozome.jgi.doe.gov/pz/portal.html#) (Goodstein et al., 2011). The PLACE database (https://sogo.dna.affrc.go.jp/) was used to analyze the *cis*-regulatory elements on the HMP family gene promoters (Higo et al., 1999).

### Plant Growth Conditions and Treatments

*Nipponbare* rice seeds (*O. sativa* L. *ssp. japonica*) were surface-sterilized with 10% (w/v) sodium hypochlorite solution for 30 min, sown onto 1/2 MS (Murashige & Skoog) solid medium, and grown in a light incubator. After 2 weeks, seedlings at the two-true-leaf stage were transplanted into Hoagland's nutrient solution and cultured under 14 h light at 28°C, 10 h dark at 22°C, and RH = 70%. *Arabidopsis thaliana* L. (Heyn) cv. Columbia plants were grown on germination medium (GM) agar plates for 2 weeks as described previously (Qin et al., 2008), transferred to vermiculite, and grown under a 16 h light/8 h dark photoperiod.

For the heavy metal treatments, the plants were exposed to 100 μM CdCl_2_, 100 μM CuSO_4_, 500 μM Pb (NO_3_)_2_, and 500 μM ZnSO_4_ (Feng, 2011; Li et al., 2015; Fu et al., 2017). Three biological replicates were prepared per treatment. The control treatment consisted of normal nutrient solution or medium. All other culture conditions were the same as those described above. The samples were harvested after treatment for 1, 3, 12, and 24 h and immediately placed in liquid nitrogen and stored at −80°C until use. The experimental procedure was repeated at least thrice.

### Expression Analysis of HMP Gene Family

To analyze the HMP gene expression profiles in different tissues, RNA-seq data were downloaded from the Expression Atlas database (https://www.ebi.ac.uk/gxa/home) (Papatheodorou et al., 2017). RNA-seq data for *Arabidopsis* used in the present research are available in the Sequence Read Archive database under accession number SRP013631 and in the GEO database under accession number GSE38612 and GSE108751. RNA-seq data for rice are available in the DNA Data Bank of Japan Sequence Read Archive under accession numbers DRR001024–DRR001051 and in the Sequence Read Archive database under accession numbers SRP008505, SRP008469, and SRP008821 and in the GEO database under accession number GSE34895. Heatmaps were generated with HemI from the normalized value by row for the signatures in transcripts per million (TPM).

Total RNA used for quantitative real-time PCR analysis was extracted from the plant tissues with TRIzol reagent (Thermo Fisher Scientific, Waltham, MA, USA) and treated with DNase I to eliminate any DNA contamination. RNA quality was assessed by gel electrophoresis and the RNA was stored at −80°C until use. First-strand cDNA (10 μL) was synthesized according to the instructions for the PrimeScript™ RT Master Mix (Takara Biomedical Technology (Beijing) Co., Ltd., Beijing, China). Quantitative real-time PCR was performed as described previously (Li et al., 2019). The gene-specific primers used in the quantitative real-time PCR are listed in Table S1.

### Statistical Analyses

Statistical analyses were completed using the Statistical Program for Social Sciences (release 19.0, SPSS Inc., IBM, www.ibm.com) and Microsoft Excel 2016.

## Results

### Genome-Wide Identification, Chromosomal Distributions and Synteny Analysis of HMP in Rice and *Arabidopsis*

Forty-six and 55 candidate rice and *Arabidopsis* HMP genes, respectively, were identified by a Hidden Markov Model for the HMA domain. All HMPs were mapped onto chromosomes and named *OsHMP01*-*OsHMP46* and *AtHMP01*-*AtHMP55* according to the gene orders on their respective chromosomes. *OsHMP26, OsHMP36, AtHMP01, AtHMP03, AtHMP14, AtHMP21, AtHMP25, AtHMP30, AtHMP37, AtHMP38, AtHMP42, AtHMP43, AtHMP53*, and *AtHMP54* had two alternative splicings, *OsHMP45, OsHMP46, AtHM46*, and *AtHMP47* had three alternative splicings, and *AtHMP52* had four alternative splicings. Characteristics of the rice and *Arabidopsis* HMPs are summarized in Table S2.

OsHMP07 was the smallest protein (69 amino acids) while OsHMP11 was the largest (1,012 amino acids). Their molecular weights range from 7.65 to 108.48 kDa and their predicted isoelectric points varied from 5.01 (OsHMP35) to 11.07 (OsHMP40). Thirty-one OsHMPs were in the nuclei, 12 in the chloroplasts, and nine in the cell membranes. AtHMP44 was the smallest protein (77 amino acids) while AtHMP51 was the largest (1,001 amino acids). The molecular weights range from 8.87 to 107.39 kDa and their predicted isoelectric points varied from 4.88 (AtHMP47) to 10.14 (AtHMP34). Thirty-nine HMPs were in the nuclei, ten in the chloroplasts, five in the cell membranes, four in the cytoplasms, and four in the mitochondria. AtHMP13 and AtHMP40 were in the cytoplasms, AtHMP24 was in the extracellular spaces, AtHMP29 was in the vacuoles, AtHMP29 and AtHMP32 were in the cell walls, and AtHMP40 was in the peroxisomes. However, the subcellular localization of HMPs in rice and *Arabidopsis* in the present study is only based on purely computational prediction, the validation *in situ* should be performed in the future research.

**Figure 1:**
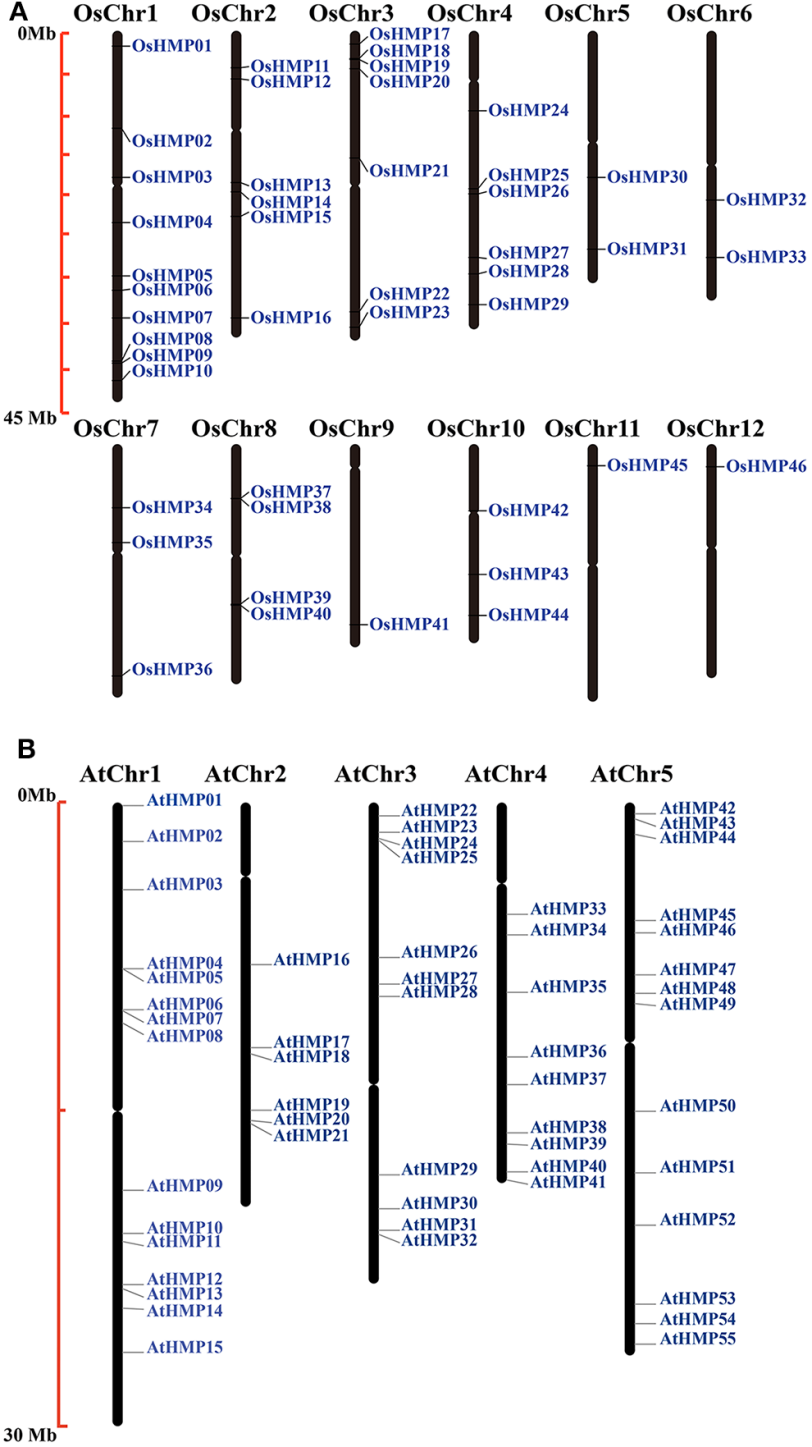
Chromosomal locations of heavy-metal-associated genes in rice **(A)** and *Arabidopsis* **(B)**. Black bars represent the chromosomes. Chromosome numbers are shown at the tops of the bar. Heavy-metal-associated genes are labeled at the right of the chromosomes. Scale bar on the left indicates the chromosome lengths (Mb).

The chromosomal locations of the *HMP*s were identified by extracting chromosomal data. Figure 1 shows that the *HMP*s were unevenly and non-randomly distributed on the chromosomes. Chr1 (chromosomal 1) contained the largest number of *OsHMP*s (10) while Chr9, Chr11, and Chr12 contained only one each. Chr1–4 contain 29 HMPs whereas Chr5–12 had only 2–3 *OsHMP*s. Thus, the *OsHMP*s were distributed mainly on Chr1–4. In contrast, the *AtHMP*s were more evenly distributed on the chromosomes. As shown in Figure 1B, the longer Chr1 and Chr5 contained more *AtHMP*s (15 and 14, respectively) while the shorter Chr2 and Chr4 contained fewer *AtHMP*s (6 and 9, respectively).

**Figure 2:**
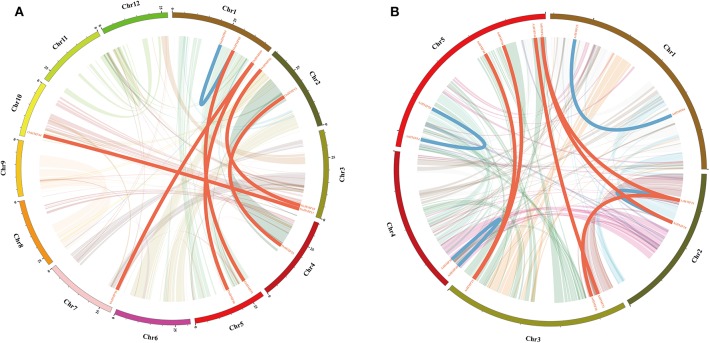
Schematic representations of segmental duplications of rice **(A)** and *Arabidopsis* **(B)** heavy-metal-associated genes. Different color lines indicate all synteny blocks in rice or *Arabidopsis* genome between each chromosome. Thick red lines indicate duplicated heavy-metal-associated gene pair on different chromosome, and thick blue lines indicate duplicated gene pair on same chromosome. The chromosome number is indicated at the bottom of each chromosome. Scale bar marked on the chromosome indicating chromosome lengths (Mb).

The *OsHMP*s had five pairs of clustered genes (*OsHMP08* and *OsHMP09, OsHMP18* and *OsHMP19, OsHMP25* and *OsHMP26, OsHMP37* and *OsHMP38*, and *OsHMP39* and *OsHMP40*). Twelve *AtHMP*s were clustered into six tandem duplication event regions on the chromosomes (*AtHMP04* and *AtHMP05, AtHMP06* and *AtHMP07, AtHMP12* and *AtHMP13, AtHMP20* and *AtHMP21, AtHMP24* and *AtHMP25*, and *AtHMP31* and *AtHMP32*). Figure 2A shows that seven segmental duplication events with 14 OsHMPs were identified and localized to duplicated segments on chromosomes 1, 2, 3, 4, 5, 7, and 10. The *Arabidopsis HMP*s exhibited eight segmental duplication events distributed on all chromosomes (Figure 2B). Therefore, synteny of the *HMP*s was highly conserved and non-diverse.

**Figure 3:**
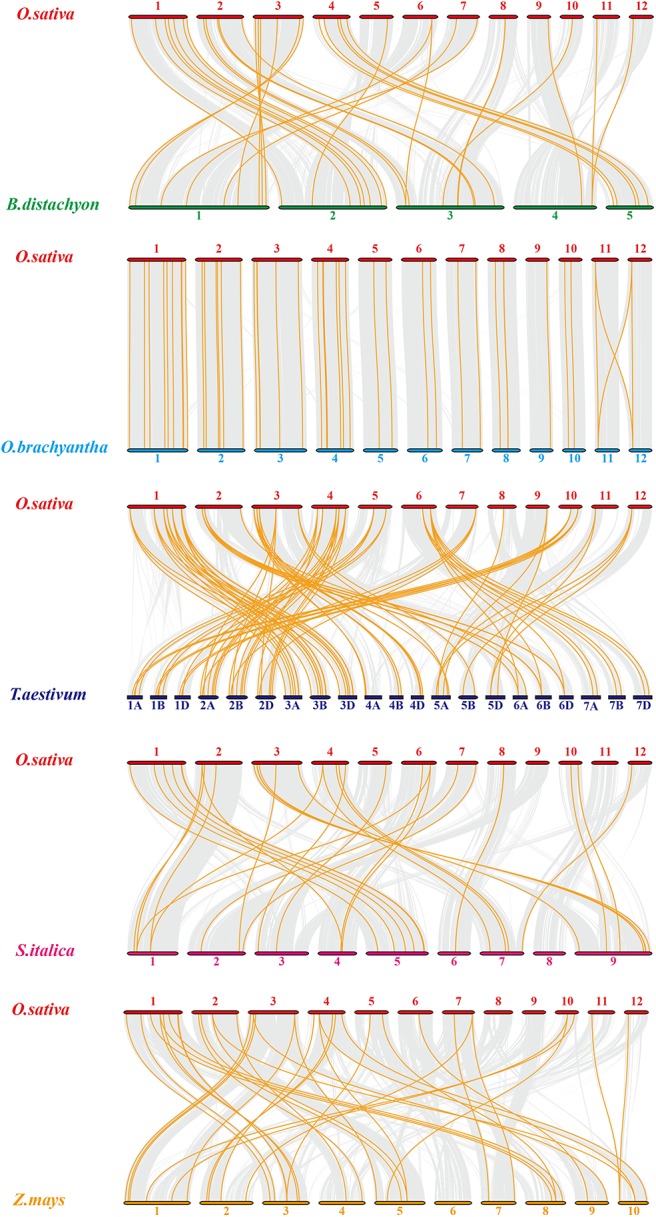
Synteny analysis of heavy-metal-associated genes between rice and *Brachypodium distachyon, Oryza brachyantha, Triticum aestivum, Setaria italica*, and *Zea mays*. Gray lines in the background indicate the collinear blocks within rice and other plant genomes, while the orange lines highlight the syntenic *OsHMP* gene pairs. The species names with the prefixes “*O. sativa*,” “*B. distachyon*,” “*O. brachyantha*,” “*T. aestivum*,” “*Setaria italica*,” and “*Z. mays*” indicate *Oryza sativa, Brachypodium distachyon, Oryza brachyantha, Triticum aestivum, Setaria italica*, and *Zea mays*, respectively. Different color bars represent the chromosomes of different species. The chromosome number is labeled at the top or bottom of each chromosome.

To expand our investigation of orthologous HMP family genes between rice and other species, we constructed 10 syntenic rice maps associated with the monocots *Brachypodium distachyon, Oryza brachyantha, Triticum aestivum, Setaria italica*, and *Zea mays* (Figure 3) and the dicots *Brassica rapa, Cucumis sativus, Glycine max, Gossypium raimondii*, and *Solanum tuberosum* (Figure S1). According to their relationships, *Oryza brachyantha* had the greatest syntenic conservation (39 HMP orthologous gene pairs distributed on all chromosomes) followed by *Zea mays* (37), *Brachypodium distachyon* (34), *Triticum aestivum* (30), and *Setaria italica* (29). Therefore, the syntenic relationships among the HMP family genes in various species are relatively conservative. Ten homologous genes between rice and soybean were detected followed by *Cucumis sativus* (2). Only one OsHMPs showed a syntenic relationship with those in *Brassica rapa, Gossypium raimondii*, and *Solanum tuberosum*. *OsHMP01, OsHMP05, OsHMP07, OsHMP09, OsHMP11, OsHMP12, OsHMP17, OsHMP18, OsHMP22, OsHMP25, OsHMP27, OsHMP28, OsHMP29, OsHMP30, OsHMP35, OsHMP43*, and *OsHMP46* were associated with all homologous gene pairs between rice and other monocots species. Therefore, these genes may have been implicated in the *OsHMP* family during gene duplication.

**Figure 4:**
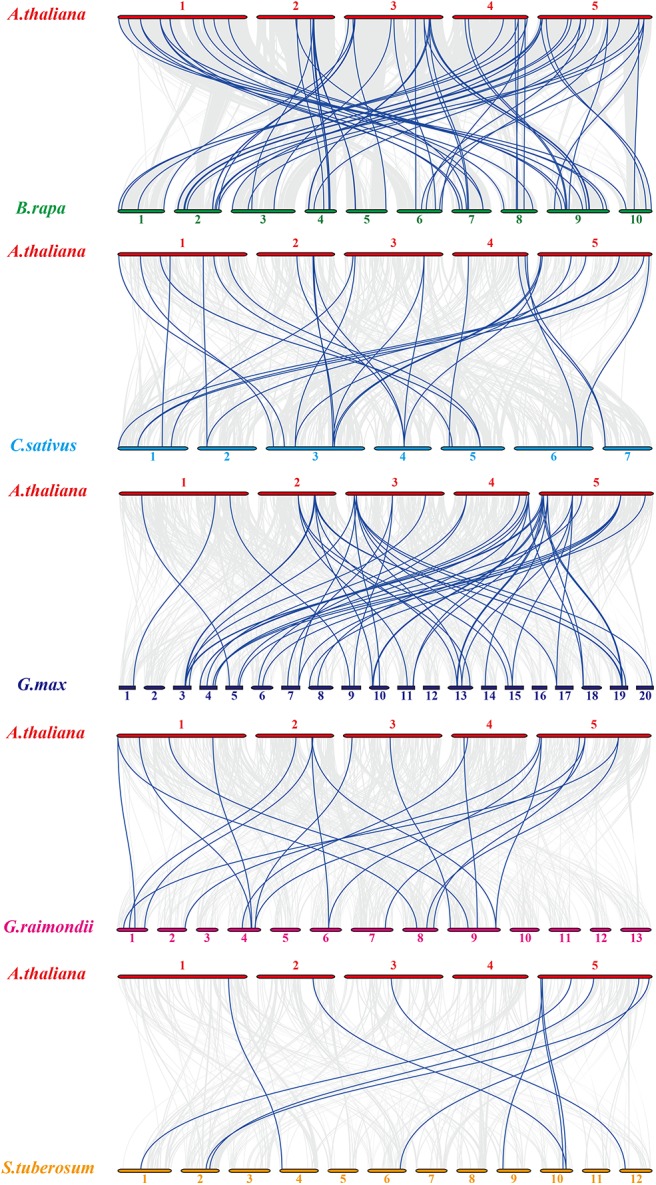
Synteny analysis of heavy-metal-associated genes between *Arabidopsis thaliana* and *Brassica rapa, Cucumis sativus, Glycine max, Gossypium raimondii*, and *Solanum tuberosum*. Gray lines in the background indicate the collinear blocks within rice and other plant genomes, while the blue lines highlight the syntenic *AtHMP* gene pairs. The species names with the prefixes “*A. thaliana*,” “*B. rapa*,” “*C. sativus*,” “*G. max*,” “*G. raimondii*,” and “*S. tuberosum*” indicate *Arabidopsis thaliana* and *Brassica rapa, Cucumis sativus, Glycine max, Gossypium raimondii*, and *Solanum tuberosum*, respectively. Different color bars represent the chromosomes of different species. The chromosome number is labeled at the top or bottom of each chromosome.

For *Arabidopsis* orthologous HMP family genes between *Arabidopsis* and other species, fifty-four *AtHMPs* showed synteny with those in *Brassica rapa* followed by *Glycine max* (49), *Cucumis sativus* (28), *Gossypium raimondii* (20), and *Solanum tuberosum* (10) (Figure 4). In addition, only three of the *AtHMPs* showed syntenic relationships with those in *Brachypodium distachyon, Setaria italica*, and *Triticum aestivum*, followed by *Oryza brachyantha* (1). No HMP gene duplication event was detected between *Arabidopsis* and *Zea mays* (Figure S2). Furthermore, only one orthologous HMP gene was detected between rice and *Arabidopsis* (Figure S3). Syntenic analysis of the HMPs of *Arabidopsis* and the other five dicotyledonous species disclosed that *AtHMP20, AtHMP42*, and *AtHMP43* were associated with ≥ 1 syntenic gene. This association may have played a central role in the gene duplication of the HMP gene family. The foregoing results show that numerous HMPs could be produced by gene replication. We calculated the Ka/Ks ratios of the HMP syntenic gene pairs to clarify the selective pressure on the HMP gene family (Tables S3, S4). Most of gene pairs had Ka/Ks < 1. However, some syntenic gene pairs showed Ka/Ks > 1, indicating that these genes might have undergone positive selective pressure.

### Multiple Sequence Alignment, Phylogenetic Analysis, and Classification of HMPs

**Figure 5:**
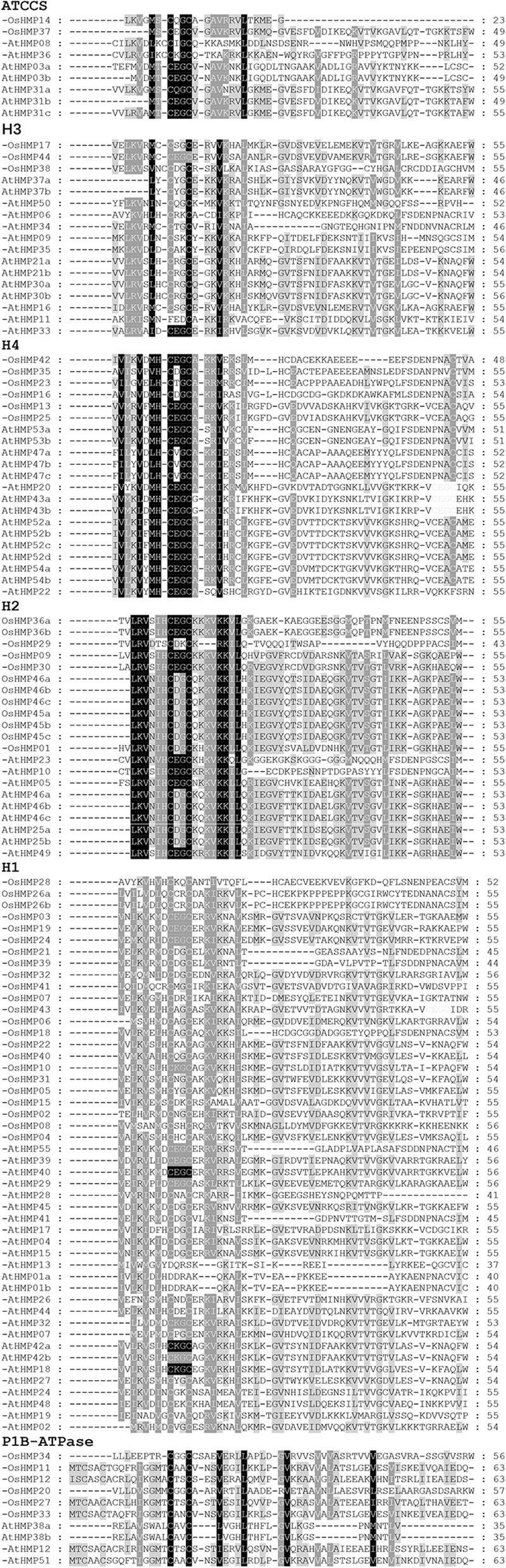
Multiple Alignment of rice and *Arabidopsis* HMP and selected heavy-metal-associated domain amino acid sequences. “ATCCS,” “H1,” “H2,” “H3,” “H4,” and “P1B-ATPase” represent different HMP proteins classification.

We examined the phylogenetic relationships of the HMPs by multiple sequence alignment of their HMA domains. Figure 5 shows that the core sequences for each classification in the HMA domain were highly conserved. Fifty-two OsHMPs and 73 AtHMPs had the highly conserved sequence “CXXC” (where “X” denotes different amino acids) while the AtHMP11 in the H3 category varied by one amino acid (“C” to “F”). For each classification, the H2 and H4 groups exhibit relatively more conservative domains. The “XX”s of the “CXXC” in their HMA domains were nearly always “DG,” “EG,” or “VG” except for “TG” in OsHMP23 in the H4 group and “DK” in OsHMP29 of the H2 group. The P1B-ATPase group presented with divergence of its HMA domains. Thus, the functions of the proteins in the P1B group may differ from those in the other groups.

**Figure 6:**
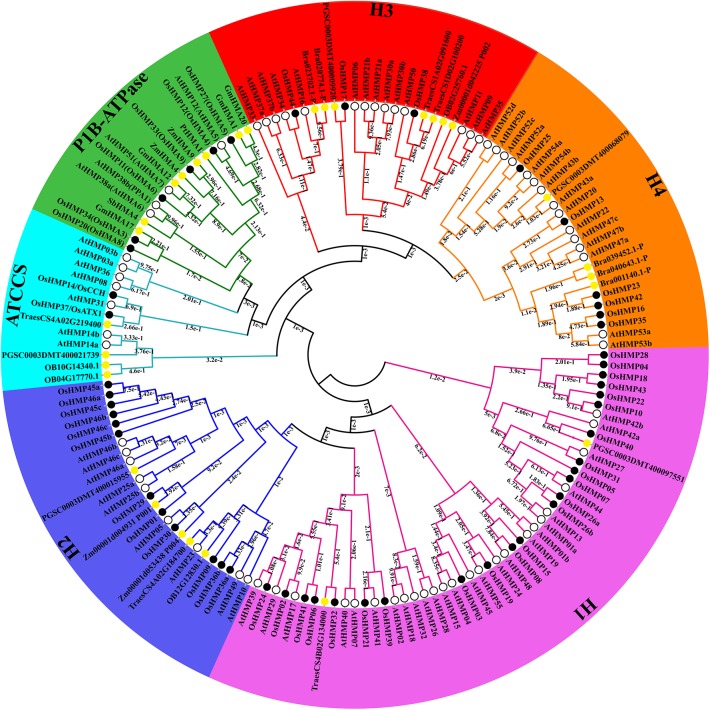
Phylogenetic relationships among 156 heavy-metal-associated proteins in rice, *Arabidopsis*, soybean, maize, potato, wheat, sorghum, wild rice, *Brachypodium distachyon*, and *Populus trichocarpa*. The maximum likelihood tree was created using MEGA v. 7.0 (bootstrap value = 1,000) and the bootstrap value of each branch is displayed. The black solid circles, hollow circles, and yellow solid circles represent heavy-metal-associated proteins from rice, *Arabidopsis*, and other species, respectively.

To expand our investigation of the phylogenetic relationships among HMPs, we built a maximum likelihood phylogenetic tree according to the results of the multiple sequence alignment of the HMA domains. Figure 6 shows that all HMPs could be divided into the subfamilies H1, H2, ATCCS, P1B-ATPase, H3, and H4 according to their structure or function. Certain genes previously identified as P1B-ATPase members including *GmHMA1, GmHMA12, GmHMA17*, and *GmHMA20* in soybean, *ZmHMA4* and *ZmHMA9* in maize, *PtHMA1* in *Populus trichocarpa*, and *SbHMA4* in sorghum were classified into the P1B-ATPase clade. The credibility of this classification was confirmed by the phylogenetic tree.

The HMA domains of the proteins in the H1 and H2 groups were located in the *N* termination. The amino acid sequences of the HMPs in the H1 clade were shorter than those in the H2 clade. The amino acid sequences in the H3 subfamily were also shorter than those in the H2 family. The HMA domains of certain proteins in the H3 subfamily were located in the *C* termination. Certain chloroplast-targeted copper chaperone proteins were detected in both the H1 and H3 subfamilies. Based on the structural differences among the HMA domains (Figure 5), the proteins in the H1 and H2 could not cluster into one group. The ATCCS clade may be implicated in metal cation transport for various peroxidases such as copper/zinc superoxide dismutase. The main function of P1B-ATPase is to help transport metal ions across biological membranes (Axelsen and Palmgren, 1998; Argüello, 2003). The biological functions of the proteins in the H4 clade, which contained two HMA domains, have not yet been established. However, it is known that the HMPs in this subfamily include isoprenylated FARNESYLATED PROTEIN 3-RELATED proteins which are involved in heavy metal detoxification and are responsive to Cd^2+^, Hg^2+^, Fe^2+^, and Cu^2+^ (Suzuki et al., 2002; Crowell and Huizinga, 2009).

### HMP Structure and Motif Composition

**Figure 7:**
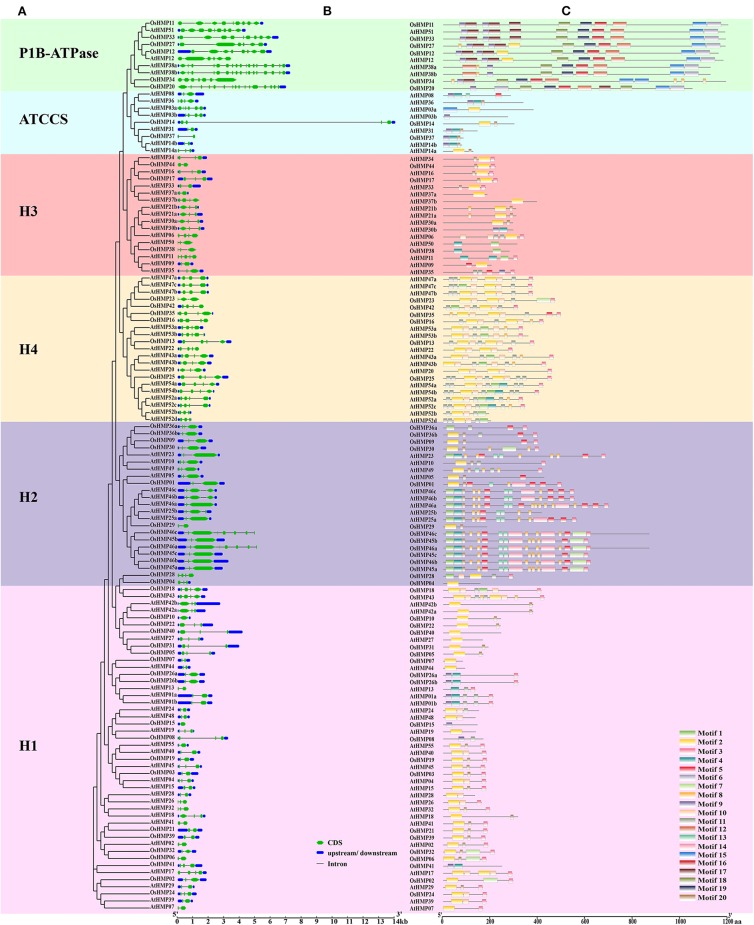
Phylogenetic analysis, gene structure and motif analysis of heavy-metal-associated genes in rice and *Arabidopsis*. **(A)** Phylogenetic tree of HMP proteins between rice and *Arabidopsis*. The maximum likelihood tree was created using MEGA v. 7.0. P1B-ATPase, ATCCS, H1, H2, H3, and H4 are marked with different colors. **(B)** Gene structure of HMP genes in rice and *Arabidopsis*. A schematic diagram was constructed by the Gene Structure Display Server 2.0. Exons, introns, and untranslated regions are marked by green double-sided wedge, black lines, and blue round-corner rectangles, respectively. The scale bar at the bottom estimates the lengths of the exons, introns, and untranslated regions. **(C)** Motif composition of HMP proteins in rice and *Arabidopsis*. Motif analysis was performed using the MEME program. Boxes of different colors represent the various motifs. Their location in each sequence is marked. Motif sequence logo is shown in Figure S4. The scale bar at the bottom indicates the lengths of the HMP protein sequences.

We analyzed the HMP gene structure to identify the differences between the HMPs from various subfamilies in rice and *Arabidopsis*. Figure 7 shows that the number of HMP exons was discontinuously distributed from 1 to 16. By combining the gene structure (Figure 7B) with the phylogenetic tree (Figure 7A), we found that the *HMP* exon numbers in various subgroups were related to their classification. All genes from the H1, H3, H4, ATCCS, and H2 clades contained 2–4 exons whereas *OsHMP46a* and *OsHMP46c* from the H2 clade contained eight exons, *AtHMP11* from the H3 clade contained five exons, and *AtHMP03a* from the ATCCS clade contained six exons. The P1B-ATPase genes in these groups contained more exons than those in other branches. Moreover, the HMPs nucleotide sequence lengths varied among different classifications. The genes of the P1B-ATPase clade had longer nucleotide sequences than those of the other groups. The nucleotide sequences of the genes in the H2 and H4 groups were longer than those in the H1, H3, and ATCCS groups but shorter than those in the P1B-ATPase subgroup except for *OsHMP14* from ATCCS whose structure may naturally vary. Homologous genes usually have similar characteristics. For this reason, the numbers of exons and the lengths of the genes are similar within the same subfamily.

We analyzed the HMP motif compositions to establish the functions of the HMP protein. We identified 20 motifs with E < 1.8 × 10^−45^. The motif sequence logo is presented in Figure S4. Motifs 1, 2, and 9 were identified as HMA domains with several altered amino acid residues. Figure 7C shows that 30 HMP proteins contained motif 1, 87 proteins contained motif 2, and 26 proteins contained motif 9. There were significant differences in the HMP protein motifs among the different classifications (Figures 7A,C). P1B-ATPase had a longer amino acid sequence than the other subgroups. It also contained specific motifs such as 6, 15, 16, 17, 18, and 19. Thus, the HMP proteins in the P1B-ATPase must have specialized physiological functions. The H4 clade had numerous short motifs and was densely distributed on the amino acid sequences. The H2 subgroup also contained several short motifs but its distribution was not as dense as that of the H4 clade. The H1, H3, and ATCCS groups all had similar motif distributions. The H1, H3, and ATCCS groups had fewer motifs and shorter amino acid sequences than the other three categories. Therefore, these proteins may have auxiliary physiological functions such as enzyme subunits or molecular chaperones.

### *HMP Cis*-Element Analysis

**Figure 8:**
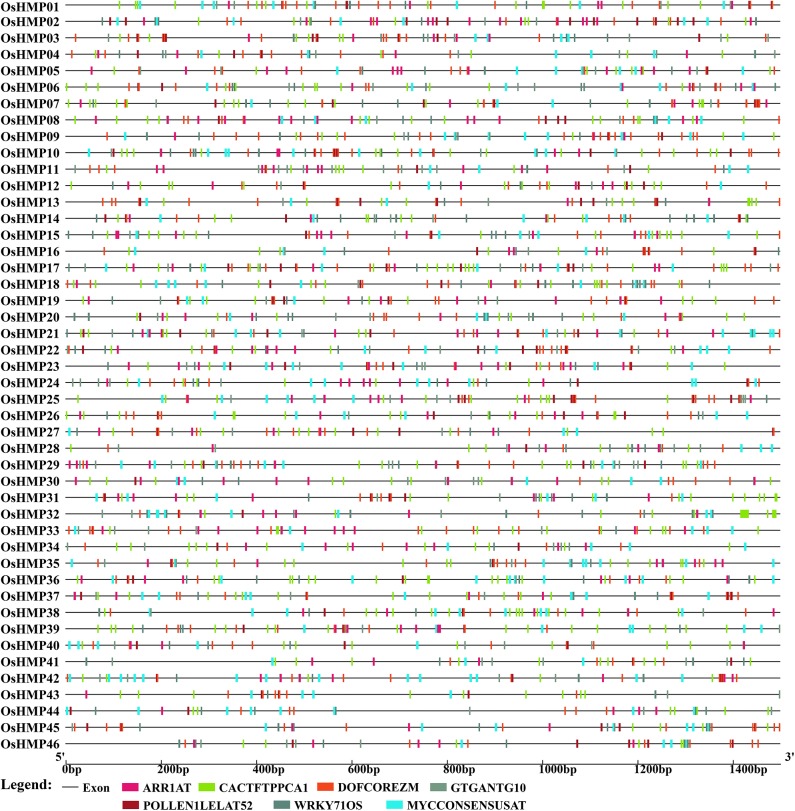
Predicted *cis*-elements in the promoter regions of the rice heavy-metal-associated genes. All promoter sequences (−1,500 bp upstream genomic sequence) were analyzed. The heavy-metal-associated genes are shown on the left side of the figure. The scale bar at the bottom indicates the length of promoter sequence. Different *cis*-elements were labeled by rectangle of different color.

To clarify upstream HMP regulation, the promoter sequences (−1,500 bp upstream of the HMP genomic sequence) of 101 rice and *Arabidopsis* HMPs were submitted to New PLACE and the *cis*-elements were investigated. In the present study, only the *cis*-elements common to all rice and *Arabidopsis* HMPs were displayed. As shown in Figure 8, seven *cis*-elements common to the *OsHMP*s were scanned out. DOFCOREZM and WRKY71OS were core sequences for transcription factor (TFs) binding (by Dof TFs and WRKY TFs) (Yanagisawa, 2000; Xie et al., 2005), ARR1AT was a type of response regulator (Ross et al., 2004), CACTFTPPCA1 was in the distal region of phosphoenolpyruvate carboxylase (Gowik et al., 2004), GTGANTG10 and POLLEN1LELAT52 were pollen-specific expression elements indicating that HMPs may participate in pollen biosynthesis (Rogers et al., 2001; Filichkin et al., 2004), and MYCCONSENSUSAT was abscisic acid (ABA) and a cold- and dehydration-responsive element. Thus, HMPs may be involved in environmental stress tolerance (Lee et al., 2005; Agarwal et al., 2006).

**Figure 9:**
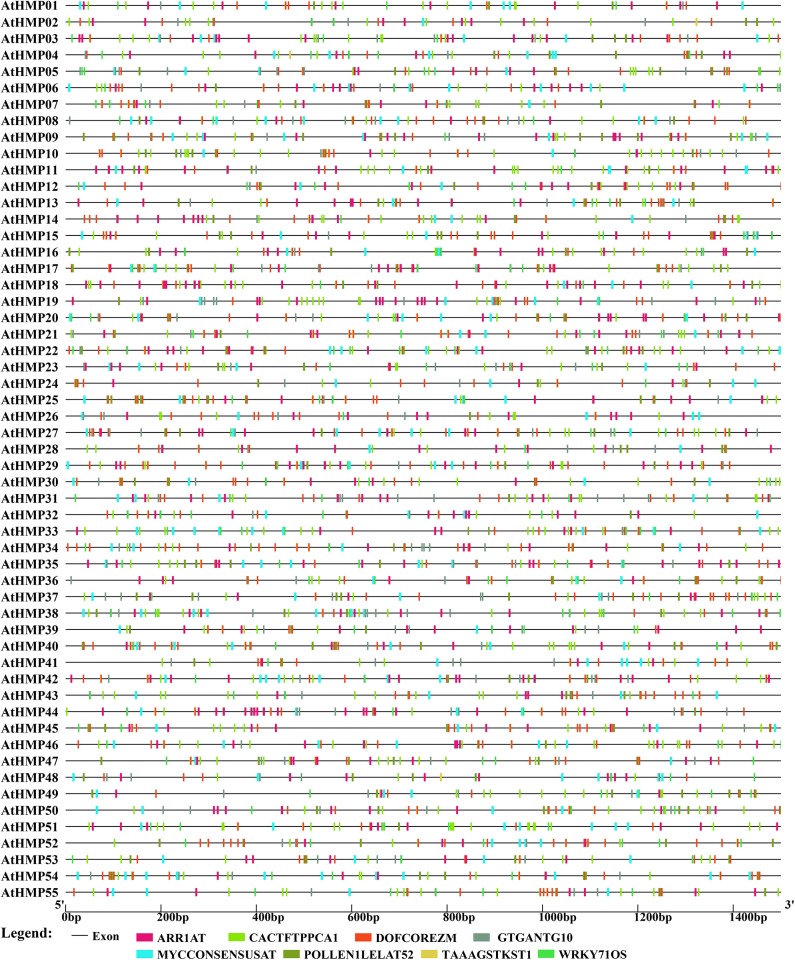
Predicted *cis*-elements in the promoter regions of the *Arabidopsis* heavy-metal-associated genes. All promoter sequences (−1,500 bp upstream genomic sequence) were analyzed. The heavy-metal-associated genes are shown on the left side of the figure. The scale bar at the bottom indicates the length of promoter sequence. Different *cis*-elements were labeled by rectangle of different color.

The *AtHMP*s contained all of the *cis*-elements detected in the *OsHMP*s (Figure 9 and Table S5). For this reason, the regulatory mechanism for HMP may not significantly differ between monocotyledons and dicotyledons. The *AtHMP*s also contained a specific *cis*-element not found in rice, namely, TAAAGSTKST1, which may be a target site for a *trans*-acting Dof protein controlling guard cell-specific gene expression (Plesch et al., 2001). The *cis*-acting element analysis indicated that the HMPs may be regulated by numerous transcription factors.

### HMP Expression Profiles in Various Rice and *Arabidopsis* Tissues

**Figure 10:**
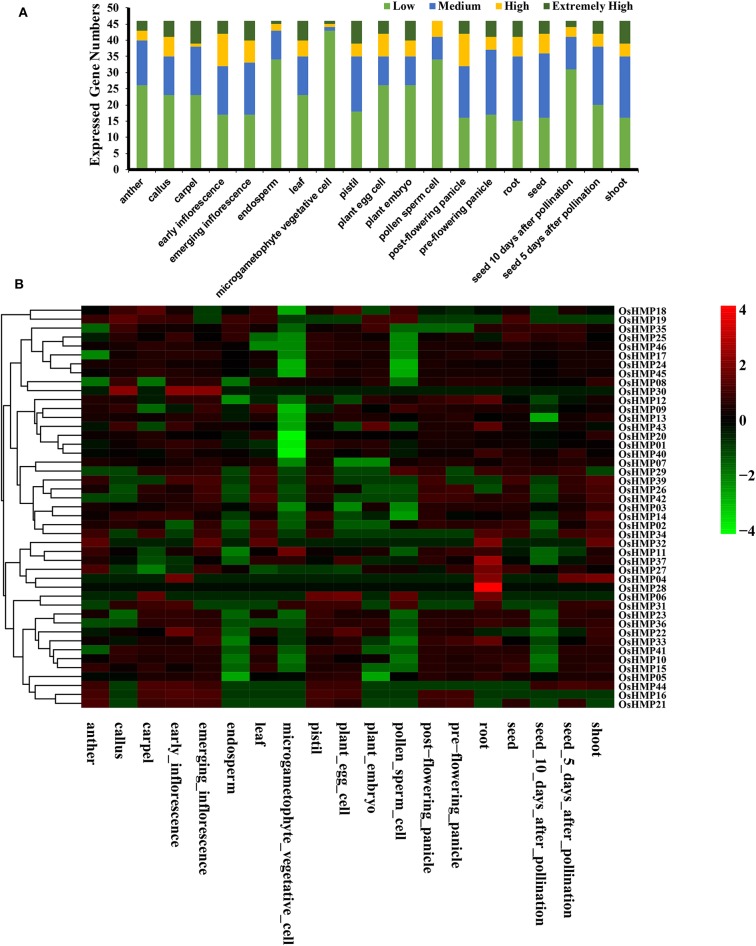
Expression pattern of the rice heavy-metal-associated gene family in various tissues or stages. **(A)** Numbers of expressed genes in each organ. Expression data of the rice HMP genes were downloaded from the Expression Atlas database. Extremely high: TPM > 100, high: 100 ≥TPM >50, medium: 50 ≥ TPM > 5, low: 5 ≥ TPM > 0; **(B)** Expression patterns of the rice HMP genes in various tissues. Heatmaps were generated using HemI from the normalized value by row for the signatures in transcripts per million (TPM). Transcript levels are depicted by different colors on the scale. Green and red represent low and high expression levels, respectively.

We evaluated the *OsHMP*s expression profiles in different tissues via the RNA-seq data (Figure 10 and Table S6). Figure 10A shows that the gene expression levels varied greatly among different tissues. Several genes were more strongly upregulated in the carpels, emerging inflorescences, leaves, pistils, embryos, and shoots than they were in the other organs. In contrast, no highly expressed genes were detected in the pollen sperm cells. Most of the *OsHMP*s in the pollen sperm cells, endosperms, and microgametophyte vegetative cells had low transcript levels.

All *OsHMP*s were expressed in ≥ 1 tissue. No pseudogenes were found in the present study (Figure 10B). Only *OsHMP28* was exclusively expressed in the root. Twelve *OsHMPs* were expressed in all 19 samples tested (TPM > 0) while eight *OsHMP*s were constitutive (TPM > 1 in all samples). *OsHMP37* (*OsATX1)* clustered into the ATCCS group had far higher transcript levels than the other seven genes in all tissues. Conversely, *OsHMP06, OsHMP16, OsHMP30, OsHMP31*, and *OsHMP41* were expressed either at low levels or not at all in every tissue. Certain *OsHMP*s preferentially expressed in specific tissues. *OsHMP04* expressed only in the roots and shoots, *OsHMP19* expressed only in the calli, *OsHMP29* expressed only in pollen sperm cells, *OsHMP32, OsHMP34* and *OsHMP36* expressed only in the roots, and *OsHMP44* expressed only in the carpels. The early inflorescences presented with higher transcript abundances than all other organs.

**Figure 11:**
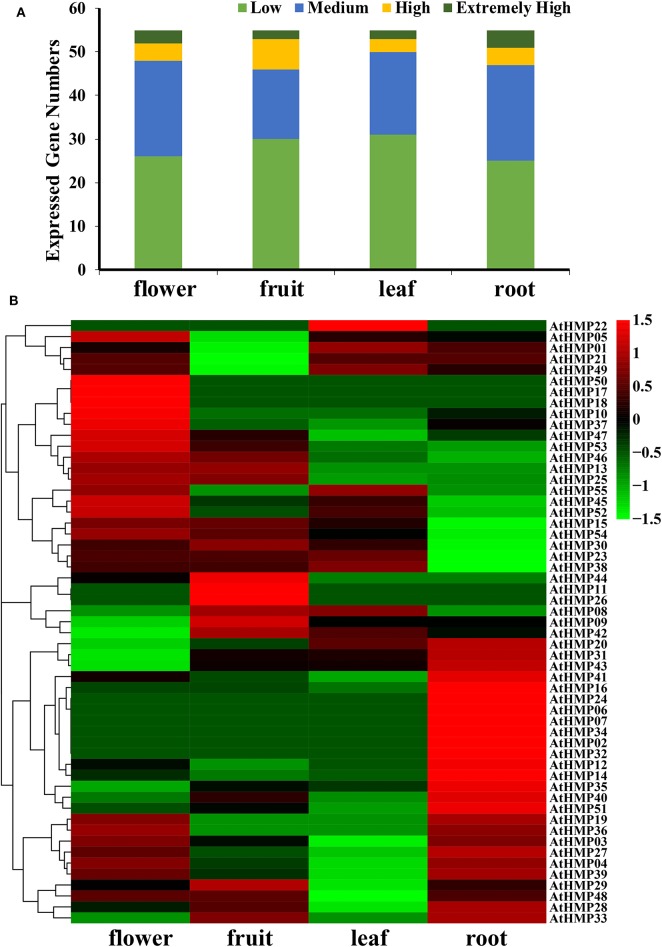
Expression pattern of the *Arabidopsis* heavy-metal-associated gene family in various tissues or stages. **(A)** Numbers of expressed genes in each organ. Expression data of the *Arabidopsis* HMP genes were downloaded from the Expression Atlas database. Extremely high: TPM > 100, high: 100 ≥ TPM > 50, medium: 50 ≥ TPM > 5, low: 5 ≥ TPM > 0; **(B)** Expression patterns of the *Arabidopsis* HMP genes in various tissues. Heatmaps were generated using HemI from the normalized value by row for the signatures in transcripts per million (TPM). Transcript levels are depicted by different colors on the scale. Green and red represent low and high expression levels, respectively.

In general, very few *AtHMP*s had extremely high transcript levels in any tissue (Figure 11A and Table S7). The *AtHMPs* exhibited tissue-specific expression while most of the *AtHMP*s maintained very low transcript levels in all tissues. Of all 55 *AtHMP*s, *AtHMP07, AtHMP08, AtHMP13, AtHMP17, AtHMP18, AtHMP22, AtHMP26, AtHMP34, AtHMP36*, and *AtHMP55* presented with low transcript levels in all tissues whereas those for *AtHMP03, AtHMP14, AtHMP20, AtHMP23, AtHMP25, AtHMP31, AtHMP35, AtHMP46*, and *AtHMP51* remained high. Certain *AtHMP*s expressed in only one tissue. *AtHMP02, AtHMP06, AtHMP24*, and *AtHMP32* expressed exclusively in the roots whereas *AtHMP11* expressed exclusively in the fruits and *AtHMP50* expressed exclusively in the flowers. *AtHMP01, AtHMP04, AtHMP05, AtHMP21*, and *AtHMP49* in the fruits, *AtHMP04, AtHMP27, AtHMP39*, and *AtHMP47* in the leaves, and *AtHMP15, AtHMP30*, and *AtHMP54* in the roots displayed low expression levels but were highly upregulated in all other tissues. In contrast, *AtHMP10* and *AtHMP37* in the flowers, *AtHMP29* in the fruits, and *AtHMP12, AtHMP16, AtHMP19, AtHMP33, AtHMP41*, and *AtHMP43* in roots exhibited high transcript levels compared to those in the other organs (Figure 11B). HMPs displayed tissue-specific expression which could indicate their particular roles in various mechanisms.

### *OsHMP* and *AtHMP* Expression Pattern Analysis in Responses to Heavy Metal Ion Stress

**Figure 12:**
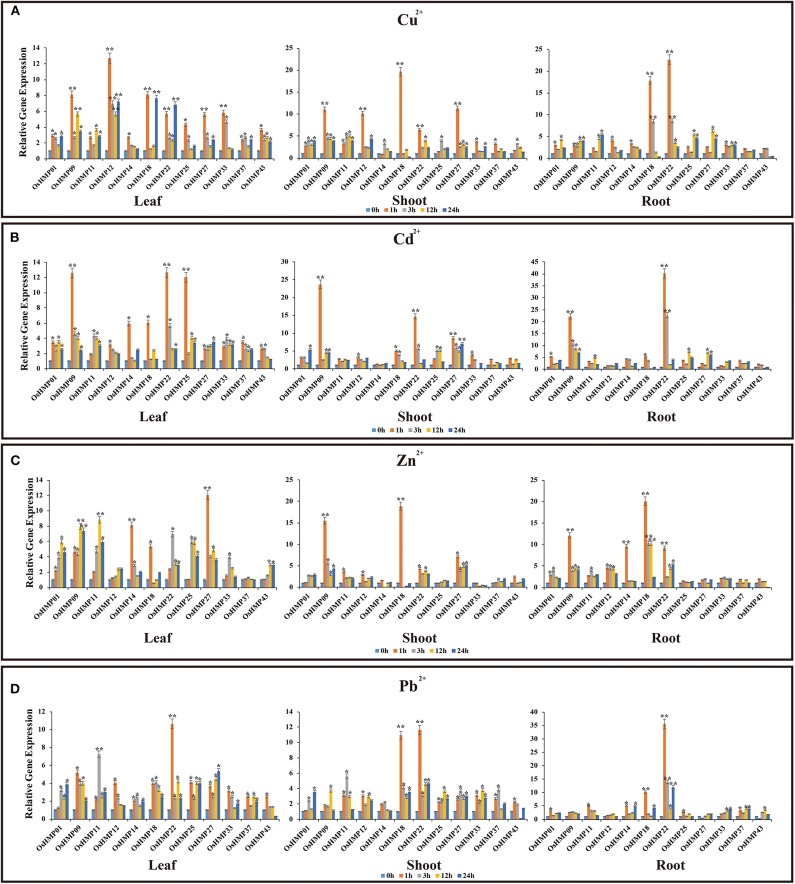
Expression profiles of 12 selected *OsHMP*s in response to Cu^2+^**(A)**, Cd^2+^**(B)**, Zn^2+^**(C)**, and Pb^2+^**(D)** stresses in 2-weeks old rice seedlings after treatment for 1, 3, 12, and 24 h. Data represent means (±SD) of three biological replicates. Vertical bars indicate standard deviations. Asterisks indicate corresponding genes significantly upregulated or downregulated between the treatment and control (*n* = 12, *p < 0.05; **p < 0.01; Student's *t-*test).

**Figure 13:**
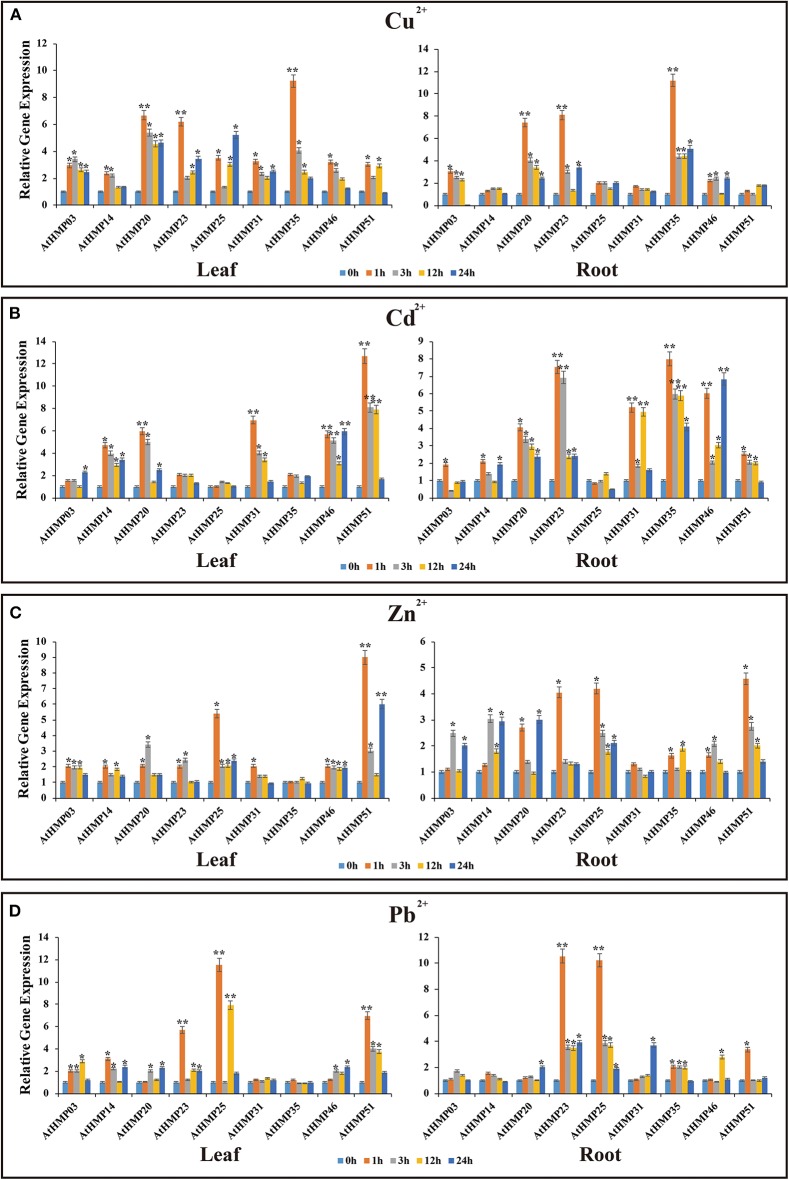
Expression profiles of 9 selected *AtHMP*s in response to Cu^2+^**(A)**, Cd^2+^**(B)**, Zn^2+^**(C)**, and Pb^2+^**(D)** stresses in 2-weeks old *Arabidopsis* seedlings after treatment for 1, 3, 12, and 24 h. Data represent means (±SD) of three biological replicates. Vertical bars indicate standard deviations. Asterisks indicate corresponding genes significantly upregulated or downregulated between the treatment and control (*n* = 12, *p < 0.05; **p < 0.01; Student's *t-*test).

We subjected rice and *Arabidopsis* seedlings to Cu^2+^, Cd^2+^, Zn^2+^, and Pb^2+^, selected 12 genes in rice and nine genes in *Arabidopsis* that were positively expressed in various organs (de Abreu-Neto et al., 2013; Xie et al., 2018), and performed qRT-PCR (Figures 12, 13). The *OsHMP* expression levels generally varied greatly under different heavy metal ion treatments in different tissues. The *OsHMP18* and *OsHMP22* expression levels under Cu^2+^ stress, *OsHMP09* and *OsHMP22* under Cd^2+^ stress, *OsHMP09* under Zn^2+^ stress, and *OsHMP22* under Pb^2+^ stress showed extremely significant difference in all tissues between the control at least one time point (Figure 12). Certain genes had stronger transcript levels in one particular tissue under specific types of heavy metal ion stress. *OsHMP09* and *OsHMP27* maintained extremely high expression levels both in the leaves and shoots under Cu^2+^ stress. Furthermore, in all tissues, *OsHMP12* was significantly and highly upregulated at all time points under Cu^2+^ stress relative to the control (Figure 12A). Moreover, *OsHMP25* in the leaves and *OsHMP27* in the shoots positively responded to Cd^2+^ stress at least one time point (Figure 12B). Under the Zn^2+^ treatment, the *OsHMP11* expression level in the leaves, *OsHMP22* in the roots, *OsHMP18* in the shoots and roots extremely significantly differed from that of the control. Although *OsHMP14* exhibited extremely significant difference between the control in the leaves and roots under Zn^2+^ stress, its transcript levels did not significantly differ from that for the control at all time points in the shoots (Figure 12C). Under the Pb^2+^ treatment, the *OsHMP11* expression level in the leaves and *OsHMP18* in the shoot and roots were extremely significantly upregulated. In contrast, neither *OsHMP14* in the shoots nor *OsHMP09, OsHMP12*, and *OsHMP27* in the roots were significantly upregulated in response to the Pb^2+^ treatment at all time points (Figure 12D).

Under heavy metal stress, the *AtHMP* transcript levels were dramatically lower than those of the *OsHMP*s (Figure 13). Overall, the relative *AtHMP* expression levels varied greatly among different heavy metal treatments. Under Cu^2+^ stress, the expression levels of *AtHMP20, AtHMP23*, and *AtHMP35* in both leaves and roots markedly differed from those for the control at least one time point (Figure 13A). Under Cd^2+^ stress, the *AtHMP31* and *AtHMP46* transcript levels in the leaves and roots, *AtHMP20* and *AtHMP51* in the leaves, *AtHMP23* and *AtHMP35* in the roots extremely significantly differed from those of the control (Figure 13B). Conversely, under the Cd^2+^ treatment, the *AtHMP25* transcript level did not significantly differ from that for the control at all time points in all tissues. Under Zn^2+^ stress, only the expression level of *AtHMP51* in the leaves showed extremely significant difference from the control at least one time point. Moreover, in the roots, nine *AtHMP*s were significantly upregulated under Zn^2+^ stress (Figure 13C). Under the Pb^2+^ treatment, the *AtHMP23* and *AtHMP25* transcript levels in both leaves and roots, *AtHMP51* in the leaves extremely significantly differed from those for the control (Figure 13D). In conclusion, these selected HMP genes are induced by at least one type of heavy metal ion, but the expression levels are different in various tissues.

In order to investigate the expression patterns of all HMPs under various heavy metal stresses, we further analyzed the expression levels in the roots under Cu^2+^ and Cd^2+^ stresses by using the transcript data from GEO database (Figures S5, S6). Sixteen of the 46 *OsHMP*s were differentially expressed (|Log2 fold change| ≥ 1) both at treatment for 1 and 3 h under Cu^2+^ stress. Fifteen of 46 *OsHMP*s were only differentially expressed at treatment for 1 h under Cu^2+^ stress. Consistent with the Cu^2+^ treatment, 14 of the 46 *OsHMP*s were differentially expressed (|Log2 fold change| ≥ 1) both at treatment for 1 and 3 h under Cd^2+^ stress, and 13 *OsHMP*s were only differentially expressed at treatment for 1 h under Cd^2+^ stress. Although *OsHMP04, OsHMP07, OsHMP13, OsHMP15, OsHMP29, OsHMP32*, and *OsHMP44* showed low expression levels under normal growth condition, these genes were upregulated under heavy metal stress (Figure 10B and Figure S5). For *AtHMP*s, 37 of the 55 *AtHMP*s were differentially expressed (|Log2 fold change| ≥ 1) under 1.3 or 1.6 μM Cu^2+^ stress, and 33 *AtHMP*s were differentially expressed under 3 or 15 μM Cd^2+^ stress. Compared with the expression profiles in various *Arabidopsis* tissues under normal growth condition, *AtHMP05, AtHMP06, AtHMP07, AtHMP09, AtHMP10, AtHMP11, AtHMP30, AtHMP32, AtHMP38, AtHMP41, AtHMP43, AtHMP50, AtHMP52, AtHMP54*, and *AtHMP55* were upregulated under heavy metal stress (Figure 11B and Figure S6). These results indicated that some HMPs expression only upregulate under heavy metal stress instead of normal growth condition, implied heavy metal-associated proteins have specific expression patterns under various heavy metal stresses.

## Discussion

Collinearity of the HMPs in rice and *Arabidopsis* has not been systematically studied. As a rule, gene synteny consists of tandem and segmental duplications (Cannon et al., 2004). Here, we detected 12 pairs of syntenic *OsHMP*s and 14 pairs of syntenic *AtHMP*s (Figure 2). Both tandem duplication (five pairs in rice and six pairs in *Arabidopsis*) and segmental duplication (seven pairs in rice and eight pairs in *Arabidopsis*) were involved in HMP collinearity. A previous study demonstrated that syntenic conservatism increased with the number of duplicated genes in the same gene family (Willis et al., 2017). The present study revealed that the synteny of HMPs are highly conservative. By comparing *Arabidopsis* and rice collinearity, we identified 17 homologous gene pairs among various rice species but only three homologous gene pairs among different *Arabidopsis* species. The collinearity differences observed between monocots and dicots is that generally synteny is maintained at a much higher level in the same species (Eckardt, 2001).

Protein structure determines its function (Aebersold and Mann, 2016). Here, alignment of the HMA domain sequences showed that the core sequences of the HMA domain in each group, namely, “CXXC,” were highly conserved (Figure 5). However, AtHMP11, OsHMP23, and OsHMP29 may have been affected by environment or genetic recombination during evolution and could have undergone natural variation. For these reasons, AtHMP11, OsHMP23, and OsHMP29 may have unique functions among the HMPs and could be applied toward population genetics or gene function research in the future.

All HMPs were divided into six clades according to the characteristics of the HMA domain (Figure 6). In a previous study, HPP and HIPP were divided into five groups and the proteins in the ATCCS group were also included (Khan et al., 2019). The latter were named in the present study. To elucidate the evolutionary relationships among the HMPs, we placed those involved in peroxidase in a separate group and divided HPP and HIPP into four groups based on their HMA domain characteristics. The highly important P1B-ATPase HMPs were also displayed in the evolutionary tree along with HPP and HIPP. Previously, they had only been investigated separately. Although certain HMPs belong to the P1B-ATPase classification, there are P1B-ATPase proteins such as *LOC_Os06g47550, LOC_Os06g48720, At4g37270, At4g30110, At4g30120*, and *At2g19110* that do not contain HMA domains (Pedersen et al., 2012; Zhiguo et al., 2018).

Subsequent gene structure and motif analyses established that genes within the same subfamilies were relatively conserved (Figure 7). However, the properties of the HMPs and their proteins varied widely under the different classifications. Thus, HMPs may be functionally diverse in plants. Comparative analysis of the HMPs in rice and *Arabidopsis* disclosed that the HMPs in *Arabidopsis* had more alternative splicing than those in rice. An earlier study revealed that the differences in the exons/introns among gene family members were indicative of their vital roles in the evolution of these genes under environmental stress (Laloum et al., 2018). Thus, *Arabidopsis* HMPs could have been subjected to more environmental stress than rice during evolution.

Determination of the *cis*-element distributions on gene promoters clarifies the signaling pathways in which HMPs are implicated. Here, it was found that the *cis*-elements common to all HMP promoters participated in transcription factor regulation and pollen-specific expression. Distribution of the HMP *cis*-acting elements resembled that for the RMP gene class, which disclosed several *cis*-elements distributed on the RMP gene promoters including ARR1AT, DOFCOREZM, GTGANTG10, POLLEN1LELAT52, and CACTFTPPCA1 (Nguyen et al., 2016). All of these were detected in the rice and *Arabidopsis HMPs*. The RMP genes are preferentially expressed in pollen. GTGANTG10 and POLLEN1LELAT52 are pollen-specific *cis*-acting elements (Nguyen et al., 2016). Therefore, the HMPs genes may play critical roles in pollen and could be focal points for future gene function studies.

Previous functional analyses of plant HMPs focused mainly focus on the mechanisms of their responses to various heavy metal ions (Hasan et al., 2017). Here, we identified ARR1AT, DOFCOREZM, and WRKY71OS as *cis*-acting elements in the HMP genes participating in transcriptional regulation. The Dof and WRKY transcription factors play roles in several abiotic and biotic stresses (Lindemose et al., 2013) and present with the attributes of zinc finger-like motifs. Several members of the WRKY and Dof families respond to heavy metal stress. *AtWRKY22, AtWRKY25*, and *AtWRKY29* in the leaves and roots of 3-wk *Arabidopsis* plants were induced by exposure to 2 μM Cu^2+^ (Opdenakker et al., 2012). An earlier study showed that Dof transcription factors were substantially upregulated under CdCl_2_ stress (Xu et al., 2019). However, another investigation reported that transcription factors are probably upregulated in response to the peroxide stress caused by heavy metal exposure (Dubey et al., 2014). On the other hand, the findings of this work suggested that the expression levels of these transcription factors also increase in direct response to heavy metal stress by binding the HMP *cis*-elements.

Gene expression specificity analyses of different plant tissues have demonstrated that these genes are implicated in heavy metal ion transport and detoxification in various organs (Liu et al., 2019). In previous studies, HIPP and HPP expression in rice and *Arabidopsis* and P1B-ATPase HMP expression in *Populus trichocarpa* were tissue-specific (de Abreu-Neto et al., 2013; Li et al., 2015). In the present study, the HMP expression levels varied widely among plant tissues. Only eight HMPs in rice and nine HMPs in *Arabidopsis* were constitutive in different tissues (Figures 10, 11). Therefore, these HMPs also participate in biological functions not related to stress response. *OsHMP37* (*OsATX1)* clustered into the ATCCS group and showed far higher transcript levels than the other genes in all tissues. Previous research indicated that *ATX1* participates in peroxide disposition and serves as a chaperone for Cu transport in plant cells (Shin and Yeh, 2012; Zhang Y. Y. et al., 2018). Nevertheless, certain genes remained at extremely low transcript level in all organs examined. *OsHMP06, OsHMP16, OsHMP30, OsHMP31, OsHMP41, AtHMP18, AtHMP22, AtHMP26, AtHMP34, AtHMP36*, and *AtHMP55* were only slightly expressed or not expressed at all in every tissue evaluated. An earlier study disclosed that the downregulation of certain genes helps conserve their ancestral functions (Qian et al., 2010). Consequently, these HMPs may have been conserved from their ancestors and are only inducible under special conditions.

Earlier research on heavy metal-associated proteins revealed that they play critical roles in metal ion distribution in plants (Li et al., 2015). In *Arabidopsis, AtHIPP06* was induced by Cd^2+^, Hg^2+^, Fe^2+^, and Cu^2+^ (de Abreu-Neto et al., 2013) while *AtHIPP26* participated in Cd^2+^ and Zn^2+^ traffic (Barth et al., 2009). *AtHIPP06* and *AtHIPP26* overexpression increased plant Cd^2+^ tolerance whereas triple knockout of *AtHIPP20/21/22* caused Cd^2+^ hypersensitivity in *Arabidopsis* (Tehseen et al., 2010). The present study investigated the expression patterns of HMPs with relatively higher expression levels in various organs under different heavy metal ion stresses. In this way, the ions transported by these HMPs in rice and *Arabidopsis* were identified. Most of the selected *OsHMP*s were differentially expressed in various tissues under metal ion stress. In contrast, most of the selected *AtHMPs* were expressed at the same levels in different tissues under heavy metal ions stress. The HMPs were involved in ion transport and responded to ≥ 1 type of ion stress. Nevertheless, few HMPs responded equally to all metal ions. Only *OsHMP11, OsHMP14, OsHMP18*, and *AtHMP23* positively responded to all four types of heavy metal cations (Figures 12, 13). However, these four genes belong to different clades (Figure 6). In *Populus*, the *PtHMA1*–*PtHMA4* phylogenetic cluster with the Zn/Cd/Co/Pb subclass of HMAs was induced by Cu and Ag (Li et al., 2015). These results suggested there may be no significant correlation between HMP classification and the types of heavy metal cations. However, the expression levels OsHMP and AtHMP are different and there was no significant correlation between gene expression and its classification. *OsHMP37* (*OsATX1*) showed higher expression levels in the roots than the leaves or shoots. *OsATX1* overexpression lowered the Cu^2+^ concentrations in the roots but raised them in the shoots (Zhang Y. Y. et al., 2018). Thus, *OsATX1* is expressed mainly in the roots and is, therefore, a root-specific stress response gene. Under normal conditions, it is constitutively expressed. Our research has preliminary analyzed the property, evolution, classification and function of HMP genes in dicotyledonous or monocotyledonous plants, which has laid a theoretical foundation for the future cultivation and breeding of those crops that are suffered from heavy metal pollution. In the future study, more molecular experiments, such as overexpression or CRISPR, can be performed on HMP genes to further verify their function.

## Conclusions

HMPs participate in numerous biological processes in plants. Here, 46 *OsHMP*s in rice and 55 *AtHMP*s in *Arabidopsis* were identified *in silico*. A gene duplication analysis showed that HMPs are conserved among various plant species and that some of them may have originated from a common ancestor. A phylogenetic analysis divided the HMPs into six subfamilies named H1–H4, ATCCS, and P1B-ATPase. The HMP gene structures and conserved domains varied greatly among clades. Nevertheless, they shared common *cis*-elements involved in regulatory transcription factors and pollen-specific expression. A gene expression profile analysis indicated that HMP expression varied substantially among different plant tissues. Only eight *OsHMP*s and nine *AtHMP*s were constitutive in various tissues. qRT-PCR analysis revealed that HMPs were induced in response to exposure to various heavy metal ions. The present study helped elucidate the biological functions of the HMPs in rice and *Arabidopsis*.

## Data Availability Statement

RNA-seq data used in the present research are available in the Sequence Read Archive database under accession number SRP013631, SRP008505, SRP008469, and SRP008821 and in the GEO database under accession number GSE38612, GSE34895, and GSE108751 and in the DNA Data Bank of Japan Sequence Read Archive under accession numbers DRR001024-DRR001051.

## Author Contributions

Conceptualization: JL. Data curation: XM. Formal analysis: JS and XL. Funding acquisition: DZ. Investigation: JW. Project administration: HZha. Resources: HL. Software: HZhe. Supervision: DZ. Writing—original draft: JL. Writing—review and editing: MZ. All authors have read, edited, and approved the current version of the manuscript.

## Conflict of Interest

The authors declare that the research was conducted in the absence of any commercial or financial relationships that could be construed as a potential conflict of interest.

## Supplementary Material

Figure S1 Synteny analysis of heavy-metal-associated genes between rice and *Brassica rapa, Cucumis sativus, Glycine max, Gossypium raimondii*, and *Solanum tuberosum*. Gray lines in the background indicate the collinear blocks within rice and other plant genomes, while the orange lines highlight the syntenic *OsHMP* gene pairs. The species names with the prefixes “*O. sativa*,” “*B. rapa*,” “*C. sativus*,” “*G. max*,” “*G. raimondii*,” and “*S. tuberosum*” indicate *Oryza sativa* and *Brassica rapa, Cucumis sativus, Glycine max, Gossypium raimondii*, and *Solanum tuberosum*, respectively. Different color bars represent the chromosomes of different species. The chromosome number is labeled at the top or bottom of each chromosome.

Figure S2 Synteny analysis of heavy-metal-associated genes between *Arabidopsis* and *Brachypodium distachyon, Oryza brachyantha, Triticum aestivum, Setaria italica*, and *Zea mays*. Gray lines in the background indicate the collinear blocks within *Arabidopsis* and other plant genomes, while the blue lines highlight the syntenic *AtHMP* gene pairs. The species names with the prefixes “*A. thaliana*,” “*B. distachyon*,” “*O. brachyantha*,” “*T. aestivum*,” “*Setaria italica*,” and “*Z. mays*” indicate *Arabidopsis thaliana, Brachypodium distachyon, Oryza brachyantha, Triticum aestivum, Setaria italica*, and *Zea mays*, respectively. Different color bars represent the chromosomes of different species. The chromosome number is labeled at the top or bottom of each chromosome.

Figure S3 Synteny analysis of heavy-metal-associated genes between *Arabidopsis* and rice. Gray lines in the background indicate the collinear blocks within *Arabidopsis* and rice genomes, while the red lines highlight the syntenic *HMP* gene pairs between *Arabidopsis* and rice. The species names with the prefixes “*A. thaliana*” and “*O. sativa*,” indicate *Arabidopsis thaliana*, and *Oryza sativa*, respectively. Different color bars represent the chromosomes of different species. The chromosome number is labeled at the top or bottom of each chromosome.

Figure S4 Motif sequences of heavy-metal associated proteins in *Arabidopsis* and rice.

Figure S5 Expression patterns of the rice HMP genes in various heavy metal stresses. Heatmaps were generated using HemI from the normalized value by row for the signatures in transcripts per million (TPM). Transcript levels are depicted by different colors on the scale. Blue and red represent low and high expression levels, respectively.

Figure S6 Expression patterns of the *Arabidopsis* HMP genes in various heavy metal stresses. Heatmaps were generated using HemI from the normalized value by row for the signatures in transcripts per million (TPM). Transcript levels are depicted by different colors on the scale. Blue and red represent low and high expression levels, respectively.

Table S1 Primers for quantitative real-time PCR.

Table S2 Detailed information of all HMP genes identified in the rice and *Arabidopsis* genome.

Table S3 Synteny rice HMP gene pairs and Ka/Ks ratios.

Table S4 Synteny *Arabidopsis* HMP gene pairs and Ka/Ks ratios.

Table S5 *Cis*-elements of HMPs in rice and *Arabidopsis*.

Table S6 RNA-seq data of rice HMP genes in different tissues.

Table S7 RNA-seq data of *Arabidopsis* HMP genes in different tissues.
