# Supplementary figures and images for: Heavy Metal Stress-Associated Proteins in Rice and Arabidopsis: Genome-Wide Identification, Phylogenetics, Duplication, and Expression Profiles Analysis
Source: Front Genet. 2020 May 8;11:477. doi: 10.3389/fgene.2020.00477 (PMC7225358; doi:10.3389/fgene.2020.00477)

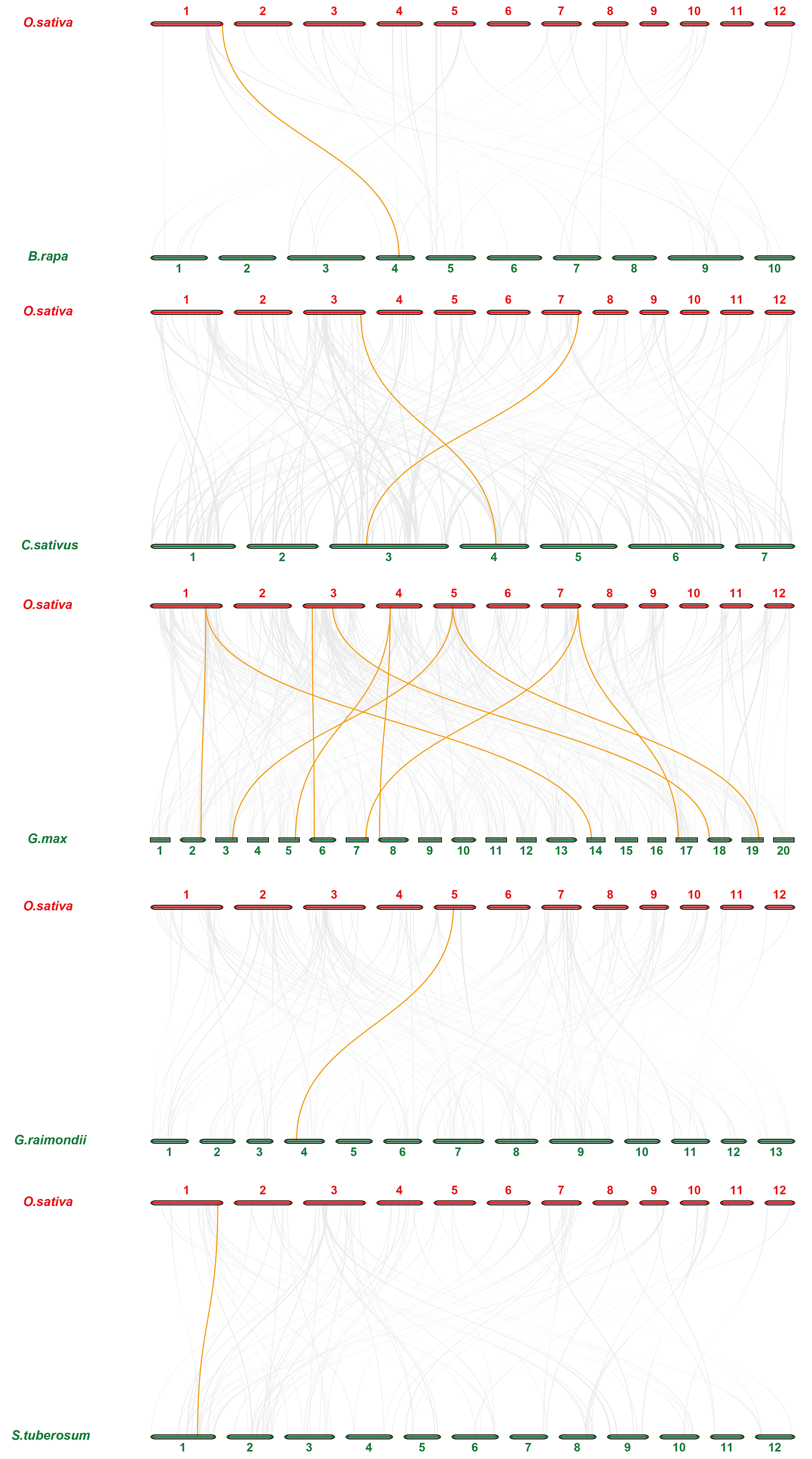

Supplement: Figure S1 — Synteny analysis of heavy-metal-associated genes between rice and Brassica rapa, Cucumis sativus, Glycine max, Gossypium raimondii, and Solanum tuberosum. Gray lines in the background indicate the collinear blocks within rice and other plant genomes, while the orange lines highlight the syntenic OsHMP gene pairs. The species names with the prefixes “O. sativa,” “B. rapa,” “C. sativus,” “G. max,” “G. raimondii,” and “S. tuberosum” indicate Oryza sativa and Brassica rapa, Cucumis sativus, Glycine max, Gossypium raimondii, and Solanum tuberosum, respectively. Different color bars represent the chromosomes of different species. The chromosome number is labeled at the top or bottom of each chromosome. [file Image_1.TIF]

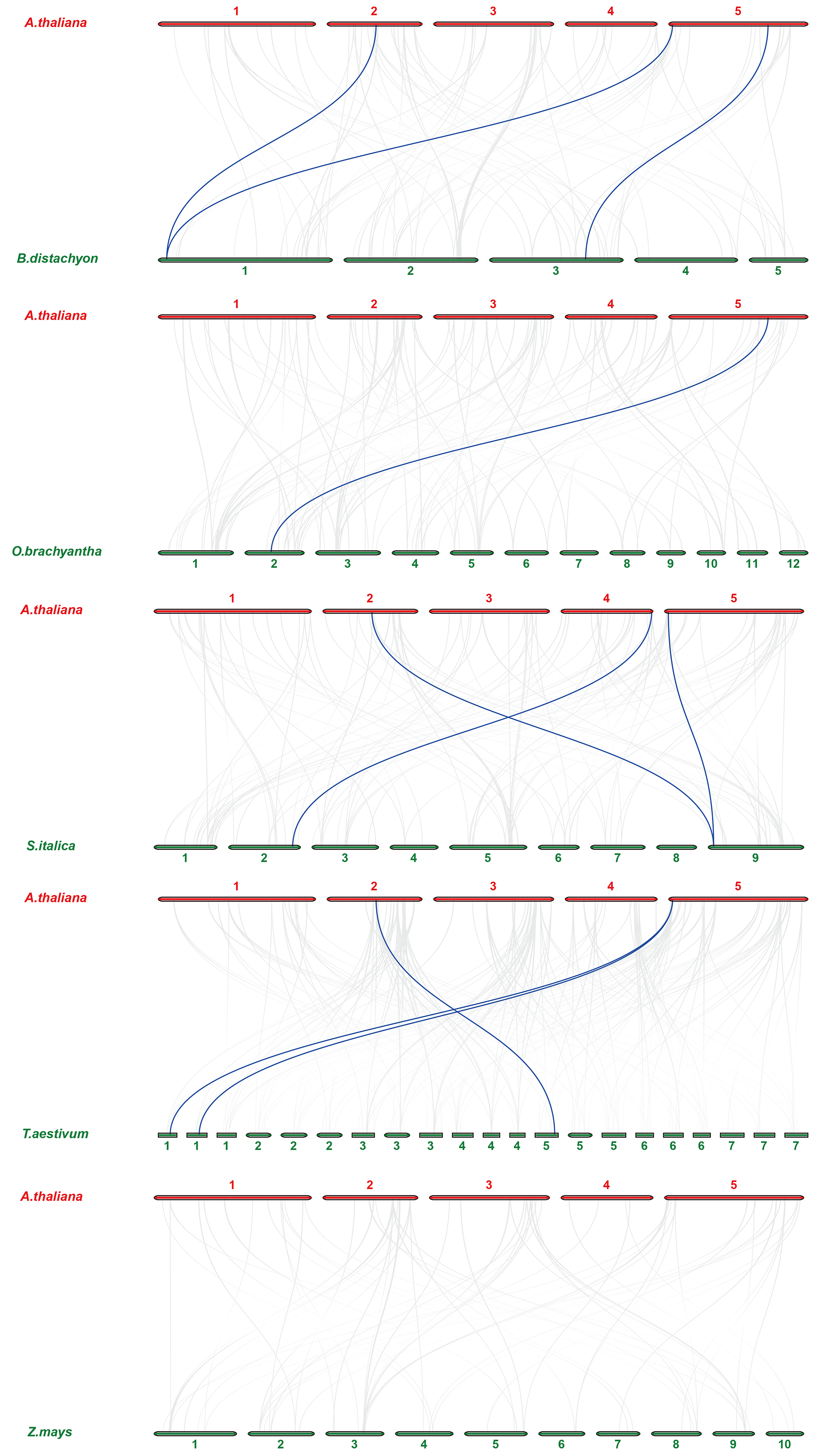

Supplement: Figure S2 — Synteny analysis of heavy-metal-associated genes between Arabidopsis and Brachypodium distachyon, Oryza brachyantha, Triticum aestivum, Setaria italica, and Zea mays. Gray lines in the background indicate the collinear blocks within Arabidopsis and other plant genomes, while the blue lines highlight the syntenic AtHMP gene pairs. The species names with the prefixes “A. thaliana,” “B. distachyon,” “O. brachyantha,” “T. aestivum,” “Setaria italica,” and “Z. mays” indicate Arabidopsis thaliana, Brachypodium distachyon, Oryza brachyantha, Triticum aestivum, Setaria italica, and Zea mays, respectively. Different color bars represent the chromosomes of different species. The chromosome number is labeled at the top or bottom of each chromosome. [file Image_2.TIF]

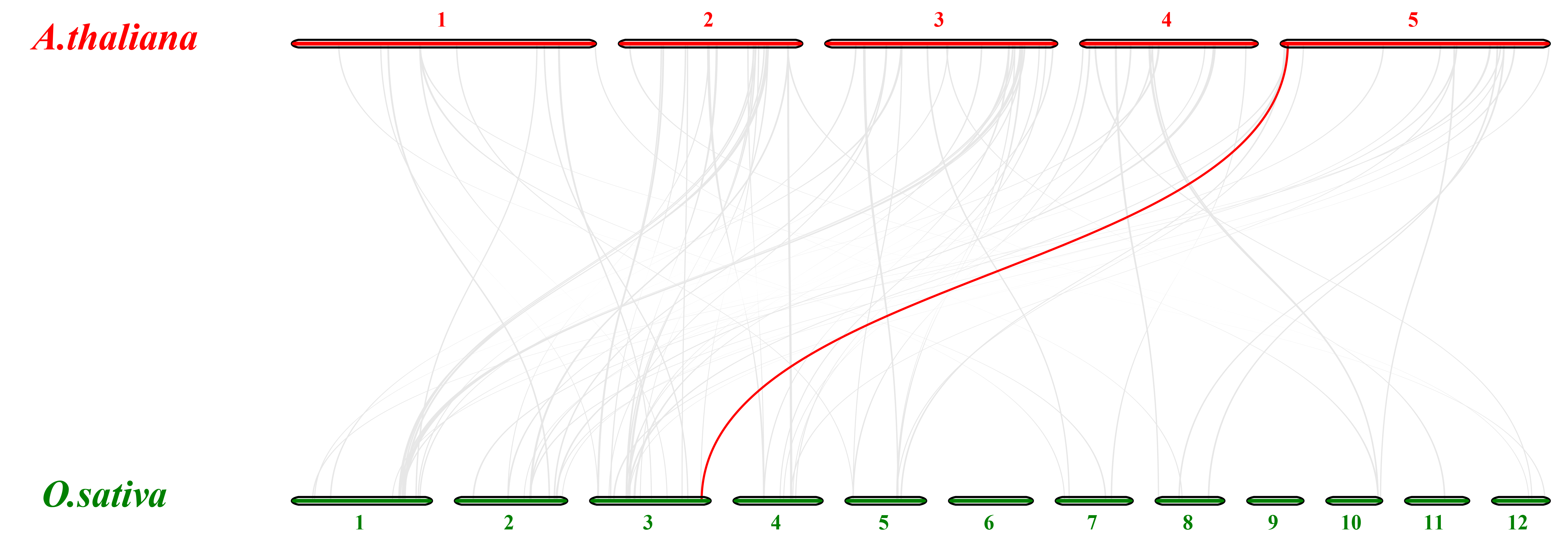

Supplement: Figure S3 — Synteny analysis of heavy-metal-associated genes between Arabidopsis and rice. Gray lines in the background indicate the collinear blocks within Arabidopsis and rice genomes, while the red lines highlight the syntenic HMP gene pairs between Arabidopsis and rice. The species names with the prefixes “A. thaliana” and “O. sativa,” indicate Arabidopsis thaliana, and Oryza sativa, respectively. Different color bars represent the chromosomes of different species. The chromosome number is labeled at the top or bottom of each chromosome. [file Image_3.TIF]

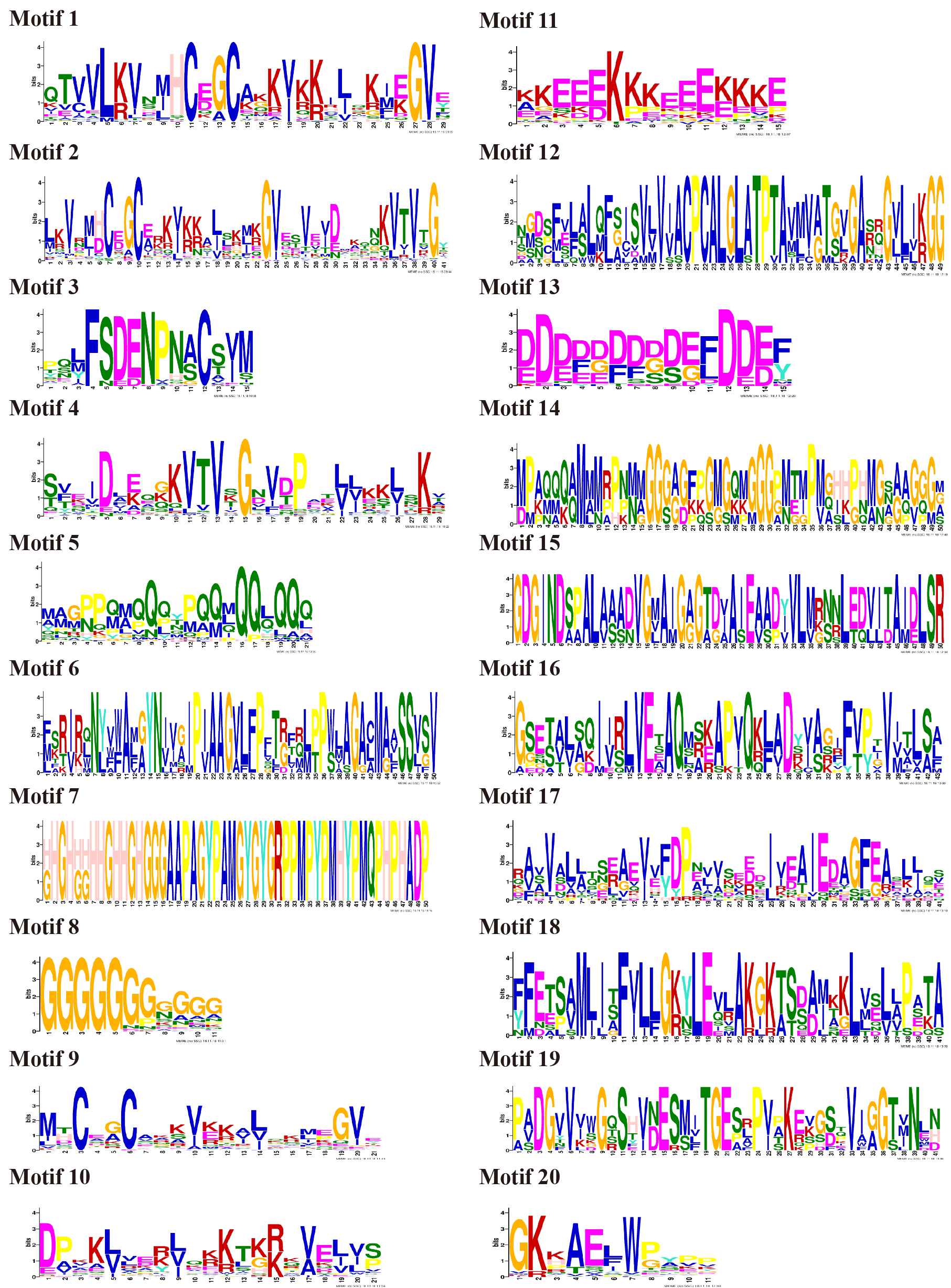

Supplement: Figure S4 — Motif sequences of heavy-metal associated proteins in Arabidopsis and rice. [file Image_4.TIF]

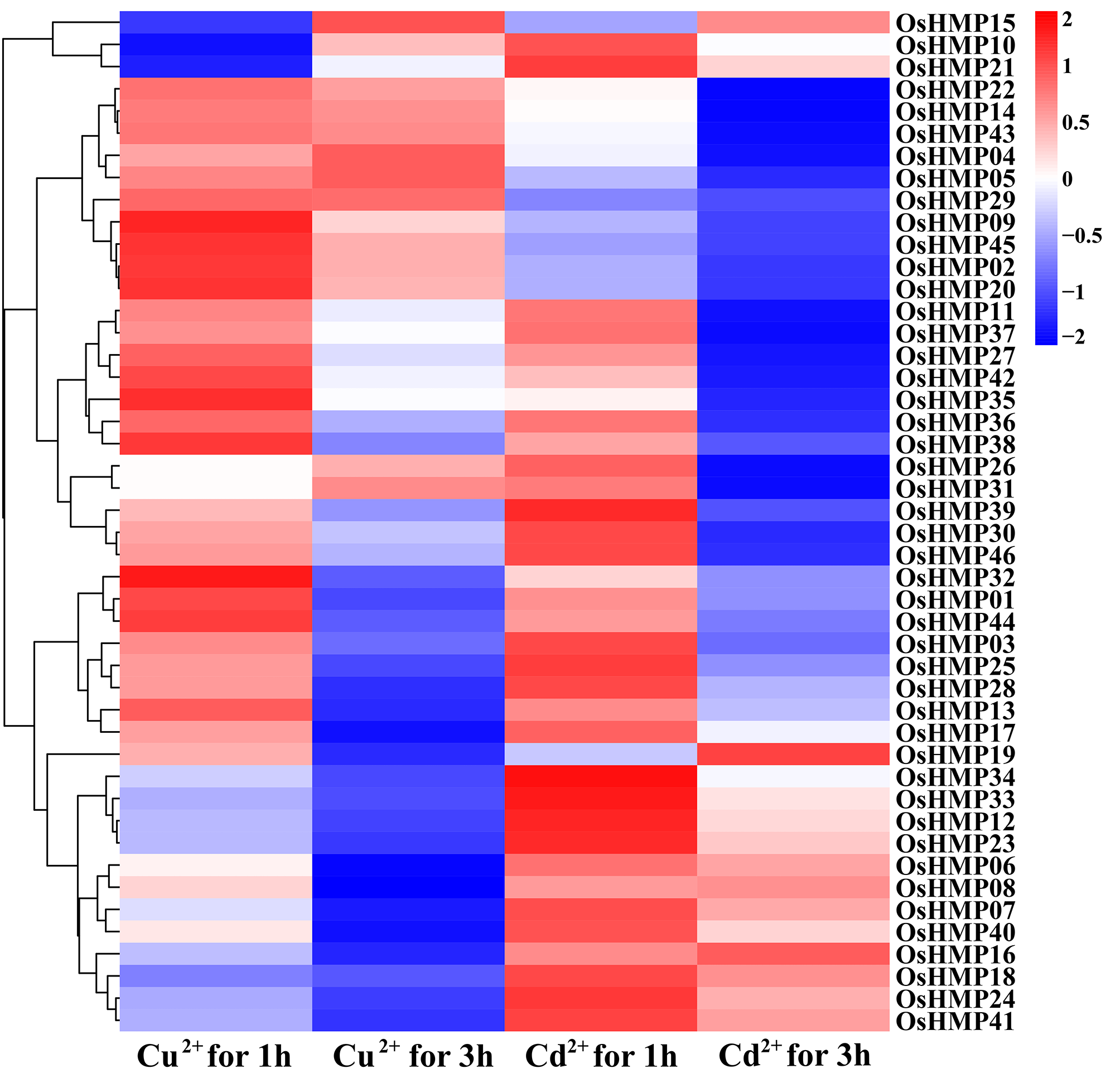

Supplement: Figure S5 — Expression patterns of the rice HMP genes in various heavy metal stresses. Heatmaps were generated using HemI from the normalized value by row for the signatures in transcripts per million (TPM). Transcript levels are depicted by different colors on the scale. Blue and red represent low and high expression levels, respectively. [file Image_5.TIF]

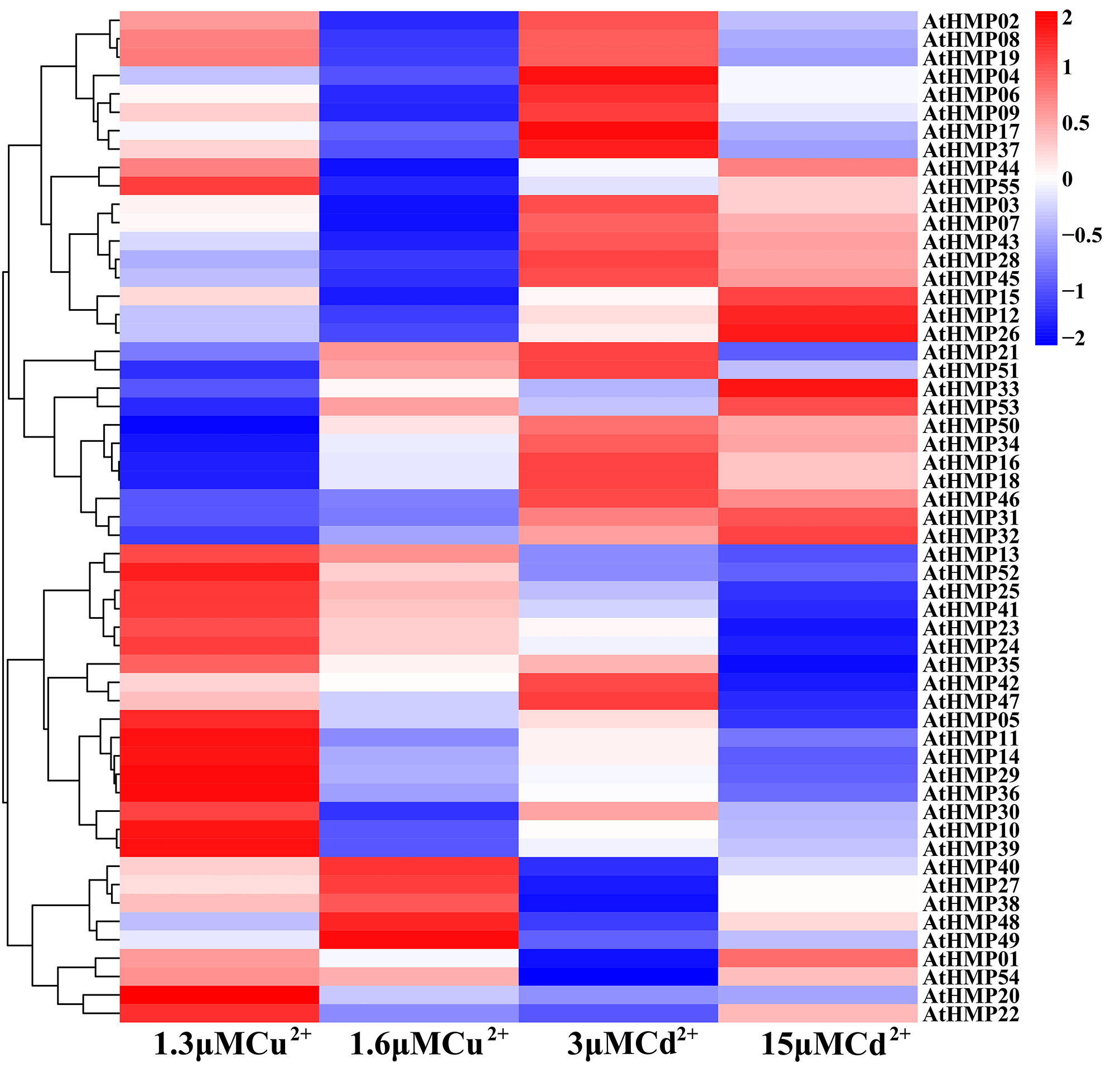

Supplement: Figure S6 — Expression patterns of the Arabidopsis HMP genes in various heavy metal stresses. Heatmaps were generated using HemI from the normalized value by row for the signatures in transcripts per million (TPM). Transcript levels are depicted by different colors on the scale. Blue and red represent low and high expression levels, respectively. [file Image_6.TIF]
